# Optimising Age‐Specific Insulin Signalling to Slow Down Reproductive Ageing Increases Fitness in Different Nutritional Environments

**DOI:** 10.1111/acel.14481

**Published:** 2025-01-24

**Authors:** Zahida Sultanova, Aykut Shen, Katarzyna Hencel, Hanne Carlsson, Zoe Crighton, Daniel Clifton, Alper Akay, Alexei A. Maklakov

**Affiliations:** ^1^ School of Biological Sciences University of East Anglia Norwich UK

**Keywords:** ageing, developmental theory of ageing, life‐history evolution, trade‐offs

## Abstract

The developmental theory of ageing proposes that age‐specific decline in the force of natural selection results in suboptimal levels of gene expression in adulthood, leading to functional senescence. This theory explicitly predicts that optimising gene expression in adulthood can ameliorate functional senescence and improve fitness. Reduced insulin/IGF‐1 signalling (rIIS) extends the reproductive lifespan of *Caenorhabditis elegans* at the cost of reduced reproduction. Here, we show that adulthood‐only rIIS improves late‐life reproduction without any detrimental effects on other life‐history traits in both benign and stressful conditions. Remarkably, we show that rIIS additively extends late‐life reproduction and lifespan when animals are exposed to a fluctuating food environment—intermittent fasting (IF)—resulting in reduced food intake in early adulthood. Full factorial genome‐wide RNA‐Seq across the life course demonstrated that IF and rIIS modulate the age‐specific expression of pro‐longevity genes. IF, rIIS and combined IF + rIIS treatment downregulated genes involved in biosynthesis in early life and differentially regulated immunity genes in later life. Importantly, combined IF + rIIS treatment uniquely regulated a large cluster of genes in mid‐life that are associated with immune response. These results suggest that optimising gene expression in adulthood can decelerate reproductive ageing and increase fitness.

## Graphical abstract

We found that reducing insulin signalling in adult worms slows down their reproductive ageing and extends their lifespan without negative side effects. Moreover, combining it with IF further enhances these benefits. These interventions likely work by regulating genes linked to biosynthesis and immunity, showing how optimising gene expression can delay ageing and improve fitness.

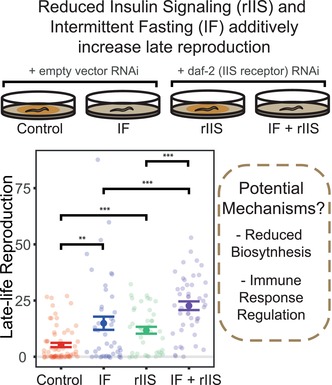

## 1 Introduction

The evolutionary theory of ageing maintains that ageing evolves because the force of natural selection declines with age (Hamilton 1966; Medawar 1952). The decline in selection gradients with age results in the accumulation of deleterious alleles whose effects on organismal performance are concentrated in late life (mutation accumulation, MA) (Medawar 1952) and favours alleles whose effects on fitness are positive in early life despite being detrimental in late life (antagonistic pleiotropy, AP) (Williams 1957). On a physiological level, there are two main proximate theories that aim to explain the evolution of ageing—the ‘disposable soma’ theory (DST) and the developmental theory of ageing (DTA) (Lemaître et al. 2024). The DST argues that ageing evolves via resource allocation trade‐off between investment into somatic maintenance, growth, and reproduction (Kirkwood 1977). The DTA proposes that ageing evolves because selection fails to optimise age‐specific gene expression, resulting in suboptimal physiology in late life (Maklakov and Chapman 2019). While the DST falls under the umbrella of AP, the DTA allows for the evolution of ageing via MA and AP alleles (Lemaître et al. 2024).

DST and DTA make unique testable predictions that can be used in empirical studies to advance our understanding of the evolutionary physiology of ageing (Lemaître et al. 2024). Specifically, the DTA predicts that optimising age‐specific gene expression using experimental approaches can postpone or slow down age‐related deterioration and improve fitness, while the DST predicts that diverting resources to improve late‐life performance will come at the cost of early‐life performance. Reproductive ageing, defined as the decline in reproductive success with advancing age, has been studied much less than actuarial ageing until recent years (Lemaître and Gaillard 2023). Here, we focused on a well‐established system of insulin signalling‐regulated life‐history in *Caenorhabditis elegans* nematodes to ask whether downregulation of insulin/insulin‐like signalling (IIS) in adulthood can slow down reproductive ageing without costs to other life‐history traits, and, therefore, improve reproductive fitness.

Previous work established that reduced IIS (rIIS) extends reproductive lifespan in *C. elegans* , but such extension was accompanied by a marked reduction in overall reproduction (Hughes et al. 2007), suggesting a genetic trade‐off. It is important to emphasise that such a genetic trade‐off does not imply direct reallocation of resources between somatic maintenance and reproduction (Luo et al. 2009). Furthermore, while rIIS improves reproductive ageing in benign environments, it is not clear whether such effects will manifest in more natural conditions when the organisms are exposed to temporary food shortages. Interestingly, different forms of food shortages, such as continuous dietary restriction (reduced intake of nutrients without malnutrition) or intermittent fasting (IF) (aka time‐restricted fasting) also can extend reproductive lifespan (Li et al. 2023; Sultanova et al. 2021). However, it is unclear whether such effects are underpinned by the same molecular pathways. For example, it has been suggested that different forms of fasting increase lifespan via different molecular signalling pathways (Greer and Brunet 2009) and the same can apply to reproductive ageing.

IF is a form of dietary restriction (reduced nutrient intake without malnutrition) that involves cycling periods of fasting and food consumption (Uno et al. 2013). Different IF regimes have been shown to extend lifespan in model organisms and reduce age‐related pathologies (Honjoh et al. 2009; Liu et al. 2019; Ulgherait et al. 2021). IF is hypothesised to act via inhibiting insulin/IGF‐1 signalling (IIS) in *C. elegans*, and previous work suggested that IF does not markedly increase lifespan in *daf‐2* mutants (Honjoh et al. 2009; Uno et al. 2013). This could be in part due to the behaviour of the DAF‐16 transcription factor, which activates longevity genes upon entering the nucleus in response to fasting but relocates back to the cytoplasm under prolonged food shortage (over 24 h) (Weinkove et al. 2006). Therefore, long‐term fasting treatments likely bypass IIS and act via different pathways, including DAF‐16 independent pathways such as AMPK (Greer and Brunet 2009; Henderson and Johnson 2001; Sun, Chen, and Wang 2017). Similarly, it is possible that different IF regimes also act via different pathways and vary in their effect on late‐life health (Greer and Brunet 2009).

Our goal was to establish whether adulthood‐only downregulation of IIS can slow down reproductive ageing and improve organismal fitness under both ad libitum and limited resources. We found that rIIS not only improves reproductive ageing to increase lifetime reproductive success in different environments, but the benefit is particularly strong when resources are limited. Then, we investigated the impact of IF and rIIS on age‐specific nuclear localisation of DAF‐16 transcription factor and found that combined IF + rIIS treatment has a stronger effect across the life course because different treatments are more effective at different ages. IF leads to DAF‐16 nuclear translocation during early adulthood while rIIS causes a more moderate DAF‐16 nuclear localisation during both early and late adulthood. Therefore, when IF and rIIS are combined, this results in a more pronounced DAF‐16 nuclear localisation from early to late‐adulthood. Finally, we investigated the effects of both treatments on genome‐wide gene expression in full‐factorial design across three different ages (young, mid‐life and old) and found that both similar and distinct physiological processes are modulated by early‐adulthood IF and rIIS treatments at different ages. Early in life, shared mechanisms, such as suppressing translation, were activated in IF, rIIS and their combination. In contrast, late in life, more distinct mechanisms, such as the ones involved in immunity, were differentially regulated by IF, rIIS and their combination.

## 2 Methods

### 2.1 Experimental Design

Three *C. elegans* wild‐type strains: N2 (Bristol, UK), JT11398 (Portugal), EG4725 (USA) and the mutant strain: *zIs356* (*daf‐16p*::*daf*‐*16a*/*b::GFP + rol*‐*6*[*su1006*]) were obtained from Caenorhabditis Genetics Center (University of Minnesota, USA, funded by NIH Office of Research Infrastructure Programs, P40 OD010440) and stored at −80°C until use. Before the setup, the defrosted *C. elegans* strains were propagated for two generations on NGM agar supplemented with nystatin (100 μg/mL), streptomycin (100 μg/mL), and ampicillin (100 μg/mL) to prevent infection, following standard recipes (Lionaki and Tavernarakis 2013). The agar plates were seeded with the antibiotic‐resistant *Escherichia coli* bacterial strain OP50‐1 (pUC4K, from J. Ewbank at the Centre d'Immunologie de Marseille‐Luminy, France) (Stiernagle 2006). To standardise parental age and eliminate any effects from defrosting, we bleached the eggs from the grandparents of experimental individuals before conducting the experiments (Stiernagle 2006).

To decrease the expression of the insulin/IGF‐1 signalling receptor homologue, *daf‐2*, during adulthood, we fed late‐L4 larvae with *E. coli* bacteria that expressed *daf‐2* double‐stranded RNA (Dillin, Crawford, and Kenyon 2002; Lind et al. 2019). As a control, we used RNase‐III deficient, IPTG‐inducible HT115 *E. coli* bacteria containing an empty vector plasmid (L4440) (Dillin, Crawford, and Kenyon 2002; Lind et al. 2019). The RNAi clones were obtained from the Vidal feeding library (Source BioScience), which was created by M. Vidal lab at Harvard Medical School, USA. Before delivery, all clones were verified through sequencing.

**FIGURE 1:**
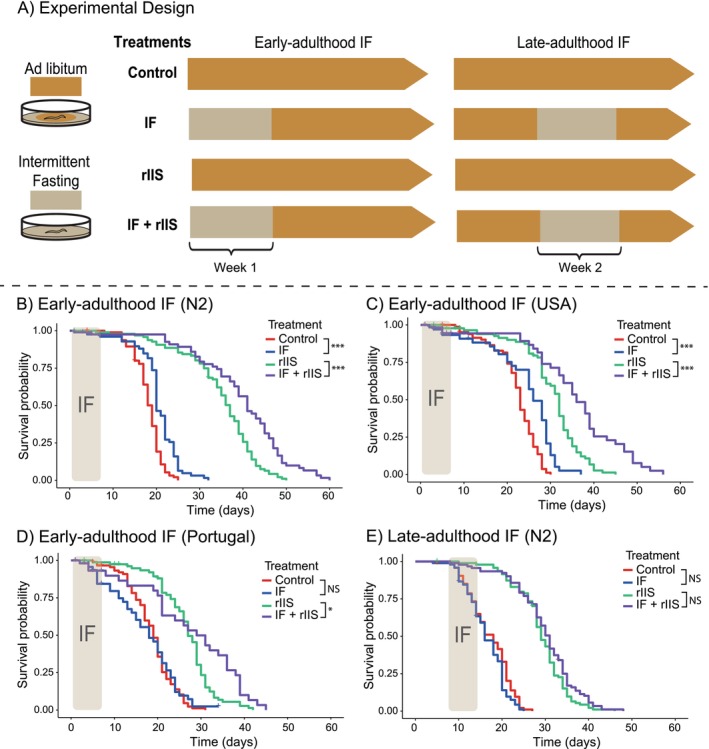
Experimental design and the effect of IF and rIIS on survival. (A) Schematic illustration of the experimental treatments. Late L4 stage worms were subjected to four different experimental treatments (Control, IF, rIIS and IF + rIIS). Early‐adulthood IF involved 9‐h fasts on alternate days during the first week of lifespan, while late‐adulthood IF followed the same schedule in the second week. Control and IF treatments were fed with *E. coli* producing empty vector RNAi; rIIS and IF + rIIS (Combined) treatments were fed with *E. coli* producing *daf‐2* RNAi. (B–D) Effect of early‐adulthood IF with and without rIIS on survival of N2, USA and Portugal worms (B, C and D respectively). (E) Effect of late‐adulthood IF with and without rIIS on survival of N2 worms. Two batches per population were used for early‐adulthood IF, and one batch for late‐adulthood IF. Survival curve divergence was assessed with log‐rank tests. NS indicates *p* > 0.05, * indicates *p* ≤ 0.05, ** indicates *p* ≤ 0.01, and *** indicates *p* ≤ 0.001.

To investigate the effects of IF and reduced insulin signalling (rIIS) on *C. elegans* survival, early and late‐life reproduction, we designed four distinct treatments (Figure 1A). ‘Control’ treatment group consisted of worms kept on standard NGM agar plates with an *E. coli* lawn that produced empty vector RNAi. ‘IF’ treatment group consisted of worms exposed to a novel IF regimen during 1 week of their lifespan by being fed with empty vector RNAi. In this IF regime, we kept worms on foodless plates for 9 h on alternate days for 1 week (i.e., on days 1, 3, 5 and 7 from the start of IF period). These foodless plates were not seeded with *E. coli* and contained no peptone, a crucial ingredient for bacterial growth. ‘rIIS’ treatment group included worms treated like control treatment but maintained on *daf‐2* RNAi‐producing *E. coli* lawn. Finally, ‘IF + rIIS (combined)’ treatment group included worms treated like IF treatment but kept on *daf‐2* RNAi‐producing *E. coli* lawn. Thus, we had a fully factorial design with two different feeding treatments and two different RNAi treatments. This approach allowed us to examine the combined effects of IF and rIIS on survival and reproduction. We applied early‐adulthood IF during the first week of lifespan to ensure the worms experienced IF for a sufficient duration without missing the reproductive period (Mendenhall et al. 2011). We applied late‐adulthood IF during the second week of lifespan because mortality starts accelerating after this period (Lind et al. 2019), and we wanted the worms to experience late‐adulthood IF before they began dying.

### 2.2 Survival Assays

To investigate the effects of IF and rIIS on worm survival, we explored how IF early and late adulthood influences the lifespan of worms in the absence and presence of rIIS. For early‐adulthood IF, we kept the worms individually during their self‐reproductive period. After reproduction ceased, we grouped the worms into cohorts of approximately 10 individuals. For late‐adulthood IF, we always kept the worms in groups until they died. We monitored survival until the death of each worm, censoring any worms that were lost during the experiment. Matricide is defined as the death of the worm due to the internal hatching of larvae and happens in < 10% of an unmated worm population. As it is different from traditional age‐related death, we showed survival without matricide in the main text. Survival including matricide is shown in Figure S1.

### 2.3 Reproductive Assays

We monitored self‐reproduction during the first week of each worm's lifespan. For the Control and rIIS treatment groups, worms were transferred to new plates every 24 h. For the IF and IF + rIIS treatment groups, worms were transferred to new plates twice on fasting days and every 24 h on ad libitum (adlib) days. To ensure that the eggs laid during the 7‐day reproductive period developed into larvae, we allowed the eggs to hatch and develop for 2 days. After this period, the hatched larvae were killed at 42°C for 3.5 h and stored for counting later.

We monitored late‐life reproduction by mating hermaphrodites with males after the self‐reproductive period ended. Males were produced by exposing around 50 L4 hermaphrodites to 30°C heat shock for 5 h on NGM plates and then incubating them at 20°C. On days 8 and 12, two males were paired with a single hermaphrodite for 24 h, after which the males were discarded. The hermaphrodites were then transferred to fresh plates daily until the cessation of reproduction on day 14. Hermaphrodites were maintained on their corresponding RNAi treatments until the end of reproduction. We replicated this experiment by maintaining all experimental worms on empty vector RNAi‐producing *E. coli* during late reproduction (days 8–14). This was done to control for any possible effect that *daf‐2* RNAi might have on male fertility and reproductive behaviour (Gems et al. 1998). This ensured that male effects were controlled for, and we report the results from this second replicate in the main text. The results from the first replicate are included in Figure S3. As matricide is important for reproduction, we showed reproduction with matricide in the main text and without matricide in Figure S3.

For intergenerational effects, we paired two males with a single hermaphrodite on day 8 for 24 h (P1 generation). We transferred the P1 worms to new plates and let them lay eggs for 24 h. We collected 50 hermaphrodite offspring (F1 generation) per treatment from these plates and maintained them on ad libitum conditions by transferring them to new plates every 24 h. For reproduction, we allowed the eggs to hatch and develop for 2 days, killed the hatched larvae at 42°C for 3.5 h and stored them for counting later. For lifespan, we monitored survival until the death of each worm and censored the ones lost during the experiment.

### 2.4 Visualisation of DAF‐16 Nuclear Localisation

We used the *zIs356* (*daf*‐*16p*::*daf*‐*16a*/*b*::*GFP + rol*‐*6*[*su1006*]) strain to observe DAF‐16 localisation. Synchronised worms expressing DAF‐16::GFP were maintained under four different treatments (control, IF, rIIS, and combined treatments; see Figure 1A) until days 1 and 7 when visualisation took place. For IF and combined treatments, visualisation occurred after a fasting period of 9–10 h. Worms were anaesthetised using 25 mM levamisole on a 2% agarose pad, with approximately 20–25 worms per slide. Data on DAF‐16 localisation were collected as binomial outcomes. A worm was classified as having nuclear GFP if at least one nucleus in its head region displayed DAF‐16‐GFP localisation; otherwise, it was classified as having cytoplasmic GFP. Worms with nuclear localisation were scored as ‘1’, based on the presence of one or more ‘filled circles’ representing DAF‐16 within the nucleus (shown by arrows in Figure 3). Worms that had a homogenous green signal without any filled circles were scored as ‘0’ (see Supporting Information for more details). This binary categorisation was chosen to quantitatively assess the influence of different treatments on DAF‐16 translocation dynamics.

### 2.5 RNA Extraction

For gene expression analysis, we collected worms from four different treatments on days 1, 7 and 13. On days 1 and 7, IF and rIIS + IF worms were collected right after the 9‐h fasting period, and the Control and rIIS worms were collected around the same time for consistency, although they did not fast. We had three biological replicates with a sample size of 100 per replicate. In total, we collected 3600 worms (4 treatments × 3 timepoints × 3 biological replicates). RNA extraction was performed separately for each replicate using TRIsure (Bioline, Cat. No. BIO‐38032) following standard phenol‐chloroform RNA extraction protocol. The purity of the isolated RNA was checked with Nanodrop, and the integrity was quantified using Agilent 2200 TapeStation System. RNA concentration was determined with Qubit using RNA HS Assay (Invitrogen Q33224).

### 2.6 Illumina RNA Sequencing

500 ng of total RNA was used to perform mRNA isolation using NEBNext Poly(A) mRNA Magnetic Isolation Module (NEB Cat. No. E7490). Isolated mRNA material was used to prepare the libraries using NEBNext Ultra II Directional RNA Library Prep Kit for Illumina (NEB, Cat No. E7760S) following the manufacturer's instructions. The resulting libraries were quantified using Qubit dsDNA HS Assay (Invitrogen Q32851).

### 2.7 Data Processing

Standard adapter sequences and common contaminants were removed from the paired‐end reads using BBduk from the BBtools package version 37.62. Positions with a Phred quality score of < 15 were trimmed, and only reads with a length > 34 were retained. The quality of the reads was assessed using FastQC version v0.12.1 and MultiQC version 1.13. The reads were aligned to the WBcel235 *C. elegans* reference genome using STAR version 2.7.8a with default parameters, and a splice junction database was generated from the Ensembl release 107 reference annotation. Gene‐level read counts were quantified using feature Counts version v2.0.3 in paired‐end mode.

### 2.8 Differential Expression Analysis

Differential gene expression analysis was performed using DESeq2 version 1.32.0. Pairwise comparisons were conducted using a Wald test, comparing different combinations of genotypes (empty vector or *daf‐2* RNAi), diets (ad libitum or IF), and time points (days 1, 7, and 13) against each other. The independent filtering feature was used to eliminate weakly expressed genes. *p*‐values were corrected for multiple testing using the false discovery rate (FDR) computed using the Benjamini–Hochberg method. Log fold change shrinkage with the ‘ashr’ shrinkage estimator was utilised to control the log_2_ fold change estimates of genes with little information or high dispersion. Differentially expressed genes were identified based on log_2_ fold change cutoffs of 1 and FDR cutoff of 0.05.

### 2.9 Gene Ontology Analysis

Gene Ontology enrichment analysis was performed separately for uniquely and commonly up‐ and down‐regulated genes using clusterProfiler version 4.2.0 together with org.Ce.eg.db version 3.17.0. *p*‐values were corrected for multiple testing using the FDR computed using the Benjamini–Hochberg method. A 0.1 (10%) FDR threshold was applied to identify enriched biological processes relative to the background. Biological processes were deemed redundant if their GO terms consisted of overlapping sets of genes and belonged to the same branch. To avoid redundancy, only the top parent terms (superset of child term) were preserved, and all child terms (subset of parent term) were filtered out from the results.

### 2.10 Statistical Analysis

Self‐fertilised reproduction—We fit generalised linear mixed‐effects models using the glmmTMB package in R, with reproduction as the response variable, and food (AL vs. IF), RNAi (empty vector RNAi vs. rIIS) and their interaction as fixed effects. We also included batch as a fixed effect and the worm id as a random effect to account for any potential batch and worm id effects. We first used different families of models including Poisson, negative binomial type 1 and 2 and generalised Poisson distribution. Then, we compared the models using Akaike information criterion (AIC) and selected the best model based on the lowest AIC score. This was done for all three populations (N2, USA and Portugal) separately.

Late‐life mated reproduction—We fit generalised linear mixed‐effects models using the glmmTMB package in R, with reproduction as the response variable, and food, RNAi and their interaction as fixed effects. We compared models with error distributions from four different families of models (Poisson, negative binomial type 1 and 2 and generalised Poisson distribution) and additional zero‐inflation parameters. We selected the best model based on lowest AIC and performed ANOVA on it.

DAF‐16 localisation—We used a generalised linear mixed‐effects model with a binomial family to analyse the relationship between DAF‐16 nuclear localisation and the interaction between RNAi, food and day, while controlling for the worm id. We used ANOVA to test for the significance of fixed effects in our glmmTMB model.

Throughout the manuscript, NS indicates *p* > 0.05, * indicates *p* ≤ 0.05, ** indicates *p* ≤ 0.01, and *** indicates *p* ≤ 0.001.

See Supporting Information for more details.

## 3 Results

### 3.1 Early‐Adulthood IF and rIIS Additively Increase Survival and Slow Down Reproductive Ageing

To understand whether IF and rIIS work through similar mechanisms as previously suggested, we explored the effects of rIIS in combination with IF. Considering that the timing of dietary interventions matters, we examined how early and late‐adulthood IF influenced survival (Figure 1 and Figure S1). We found that rIIS and early‐adulthood IF additively increased survival in the wild‐type N2 strain (Figure 1B). We also assessed the impact of rIIS and early‐adulthood IF on various strains of *C. elegans* to evaluate the generalisability of our findings beyond N2, the commonly used laboratory strain. For USA strain, early‐adulthood IF increased survival both in the absence and presence of rIIS (Figure 1C). For Portugal strain, there was no effect of early‐adulthood IF on the survival of control worms, but it further increased survival in the presence of rIIS (Figure 1D). We conducted the late‐adulthood IF experiment only on the N2 strain due to its clear and pronounced lifespan extension response to both IF and rIIS. In contrast to early‐adulthood IF, late‐adulthood IF did not affect survival, regardless of whether rIIS was present or not (Figure 1E). In summary, our findings showed that rIIS increases survival when combined with early‐adulthood IF consistently across different worm strains. Moreover, the timing of IF is important for its effect on lifespan, with early‐adulthood IF having a significant impact, unlike late‐adulthood IF (the results remained similar when matricides were included, see Figure S1 and Table S1).

**FIGURE 2:**
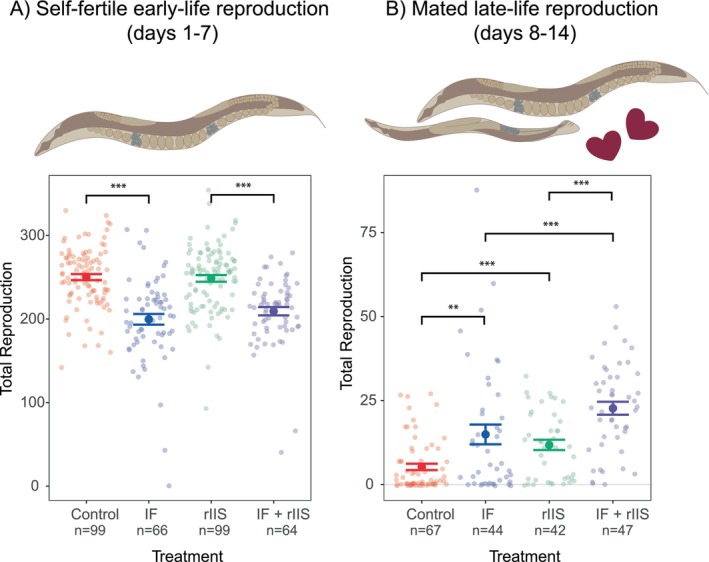
Effect of IF and rIIS on reproduction. (A) Early‐life self‐fertile reproduction. As rIIS had no effect on early‐life reproduction, IF effects were tested by performing Student's *t*‐tests for pairwise comparisons (Control vs. IF and rIIS vs. IF + rIIS). (B) Late‐life mated reproduction. Differences between treatments were assessed by Dunn test with Benforini‐Hochberg correction. Two batches per population were used for early reproduction, and one batch for late reproduction. NS indicates *p* > 0.05, * indicates *p* ≤ 0.05, ** indicates *p* ≤ 0.01, and *** indicates *p* ≤ 0.001.

Knowing that various dietary restriction types and rIIS mutants can reduce reproduction, we investigated how IF and rIIS affect self‐fertilised reproduction. IF significantly reduced reproduction (Figure 2A, IF: *χ* ^2^ = 76.41, df = 1, *p* < 0.001), whereas rIIS (via *daf‐2* RNAi) had no effect on reproduction in the N2 strain (rIIS: *χ* ^2^ = 0.65, df = 1, *p* = 0.421; interaction between IF and rIIS: *χ* ^2^ = 2.41, df = 1, *p* = 0.121) similar to previous findings (Dillin, Crawford, and Kenyon 2002; Lind et al. 2019). These effects did not change when matricides were censored and were similar in two other *C. elegans* strains (Figure S2). In brief, we showed that, in contrast to rIIS, IF decreases reproduction during its application, highlighting that dietary interventions and reducing nutrient signalling pathways can have different impacts on reproduction.

To test whether IF and rIIS slow down reproductive ageing, we explored the effects of IF and rIIS on late‐life mated reproduction in the N2 strain. Our study revealed that both IF and rIIS independently led to increased late‐life reproduction (Figure 2B, IF: *χ* ^2^ = 16.5049, df = 1, *p* < 0.001; rIIS: *χ* ^2^ = 6.9117, df = 1, *p* = 0.009; interaction between IF and rIIS: *χ* ^2^ = 0.0122, df = 1, *p* = 0.912). When IF and rIIS were combined, late‐life reproduction was higher than all other treatments (Figure 2B and Figure S3). Altogether, these data emphasise that IF and rIIS additively delay reproductive ageing, indicating their potential as interventions for extending reproductive health.

Finally, we tested whether IF and rIIS have effects that pass down to the next generation by looking at the survival and reproduction of offspring produced later in life in the N2 strain. Our findings show that neither IF nor rIIS changed how well these offspring survived (Figure S4). Similarly, there was no effect of IF or rIIS on offspring lifetime reproductive success (Figure S5, IF: *χ* ^2^ = 0.8584, df = 1, *p* = 0.354; rIIS: *χ* ^2^ = 2.5170, df = 1, *p* = 0.113; interaction between IF and rIIS: *χ* ^2^ = 0.6178, df = 1, *p* = 0.432). These results imply that the beneficial effects of IF and rIIS may not extend to the next generation.

### 3.2 Both IF and rIIS Enhance DAF‐16 Nuclear Localisation But in an Age‐Specific Manner

**FIGURE 3:**
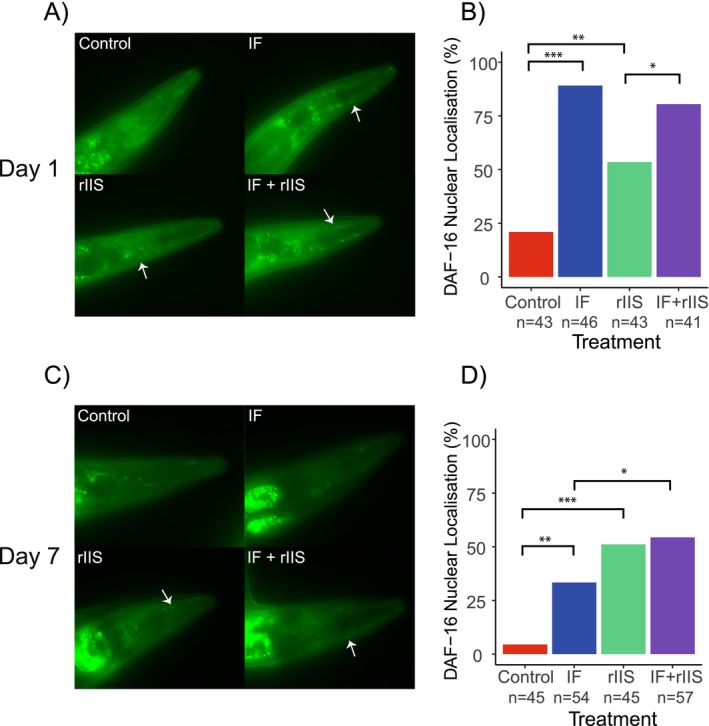
Effect of IF and rIIS on DAF‐16 nuclear localisation. (A–D) Photographs and bar plots showing the effect of experimental treatments on GFP‐tagged DAF‐16 nuclear localisation, assessed on day 1 (panels A and B) and day 7 (panels C and D). The differences between treatments were evaluated using chi‐square tests with Benforini‐Hochberg correction. NS indicates *p* > 0.05, * indicates *p* ≤ 0.05, ** indicates *p* ≤ 0.01, and *** indicates *p* ≤ 0.001.

**FIGURE 4:**
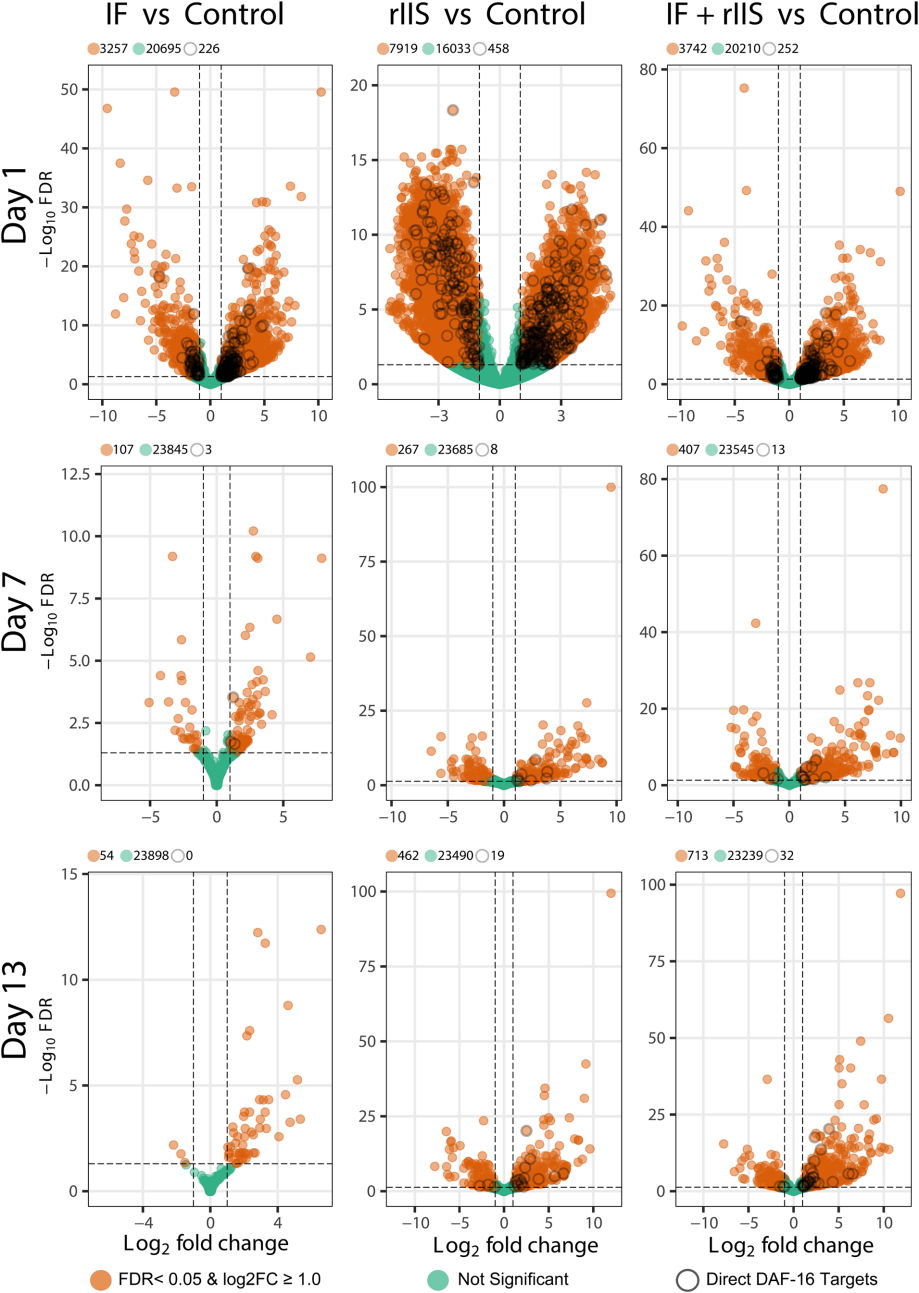
Effect of IF, rIIS, and IF + rIIS on age‐specific gene expression. Volcano plots comparing differentially expressed genes in IF, rIIS and IF + rIIS treatments versus control on days 1, 7, and 13. Direct DAF‐16 targets were taken from a previous study that identified them by using chromatin profiling by DNA adenine methyltransferase identification, DamID (see Schuster et al. 2010).

To understand the potential mechanisms underlying the effects of IF and rIIS on ageing, we explored the nuclear localisation of the DAF‐16 transcription factor. DAF‐16 activates longevity genes when it moves into the nucleus (Sun, Chen, and Wang 2017). DAF‐16 localisation was explored on days 1 and 7 but not on day 13 for three reasons. First, early‐adulthood IF, which led to lifespan extension, was only applied during the first week of lifespan. Second, DAF‐16 nuclear localisation assays of the IF treatments were performed after a 9‐h fasting period which ended on day 7 for the early‐adulthood IF treatment. Third, for reproductive ageing, worms were mated right after day 7 and then they were all kept under the control condition (no IF/rIIS). So, the DAF‐16‐related factors that modulate reproductive ageing are expected to occur in the first week of lifespan. We observed that IF significantly increased DAF‐16 nuclear localisation during early‐adulthood (Figure 3A,B), but this effect diminished gradually with advancing age (Figure 3C,D). On the other hand, rIIS also caused DAF‐16 to move into the nucleus, but this effect was weaker compared to IF during early‐adulthood (Figure 4A,B). Interestingly, unlike IF, rIIS continued to enhance DAF‐16's localisation into the nucleus as worms grew older (Figure 3C,D). These varying effects at different ages led to a significant interaction between IF and rIIS (*χ* ^2^ = 5.12, df = 1, *p* = 0.02) with marginally nonsignificant interactions between rIIS and age (*χ* ^2^ = 3.18, df = 1, *p* = 0.07) and between IF and age (*χ* ^2^ = 3.10, df = 1, *p* = 0.08). Thanks to the early‐life effect of IF and the sustained impact of rIIS during both early and late life, the combined treatment benefited from higher DAF‐16 nuclear localisation throughout the lifespan (Figure 3), potentially enhancing longevity and late‐life reproduction.

### 3.3 IF, rIIS and Their Combined Treatment Exert Age‐Dependent Effects on Gene Expression

We analysed gene expression changes in worms subjected to IF, rIIS or both on day 1, 7 and 13 by sequencing polyA(+) RNA in triplicate for each condition and time point. The principal component analysis (PCA) of samples shows that the largest variation in gene expression changes is due to the ageing of animals, as PC1 can separate all Day 1 animals from Day 7 and Day 13 (Figures S6–S8). Next, we calculated the differential gene expression changes in worms exposed to IF, rIIS and combined treatments compared to control worms (Figure 4). The largest gene expression changes are observed in day 1 animals, and the number of differentially expressed genes between conditions go down as the animals age for day 7 and 13 samples. Moreover, while rIIS treatment resulted in the highest number of differentially expressed genes in the early life, the combined treatment led to a greater number of differentially expressed genes at more advanced ages (Figure 4). This shows that when IF and rIIS are combined, their effect on gene expression can further extend to older ages.

Next, to understand how DAF‐16 activation contributes to the differential gene expression across treatments and time points, we analysed the presence of previously identified direct targets of DAF‐16 (Schuster et al. 2010). Direct DAF‐16 targets behaved similar to the total number of differentially expressed genes (Figure 4). During early adulthood (day 1), most of the direct DAF‐16 target genes were upregulated by rIIS. As the worms aged (days 7 and 13), rIIS alone was not enough and the combination of IF and rIIS was necessary to upregulate many DAF‐16 direct targets (Figure S9A). The pattern was similar for downregulated DAF‐16 targets, although fewer targets were affected (Figure S9B). These findings further suggest that the combination of IF and rIIS is necessary to maintain the activation of ageing related target genes in older worms.

### 3.4 IF, rIIS and Their Combination Have Age‐Specific Effects on Diverse Biological Functions, Particularly Those Related to Biosynthesis and Immune Response

We conducted a Gene Ontology analysis to explore the functions of genes differentially expressed with age across different treatments. First, we looked at genes differentially expressed in the combined IF + rIIS treatment compared to the control to understand which genes are affected by the combination of IF with rIIS (Figure S10). During early adulthood (day 1), the combined treatment enhanced the expression of genes involved in developmental processes and simultaneously reduced the expression of genes associated with peptide metabolism (Figure S10A), this was similar to both IF (Figure S11) and rIIS (Figure S12) treatments. Conversely, in late adulthood (days 7 and 13), distinct groups of genes associated with immune response exhibited varied expression patterns, some upregulated, and others downregulated (Figure S10B and S10C). As the worms aged, the GO terms of the combined treatment became more similar to rIIS treatment (see Figure S12). These findings suggest that the combined treatment modulates gene expression differently at different ages, suppressing biosynthesis in early adulthood (because of both IF and rIIS) and altering immune response genes in late adulthood (mostly due to rIIS).

**FIGURE 5:**
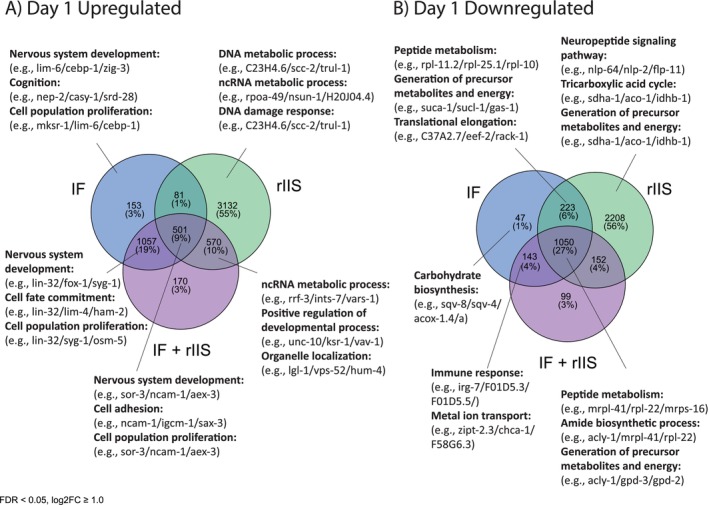
Gene Ontology analysis of differentially expressed genes across IF, rIIS and IF + rIIS treatments on day 1. GO terms showing the functions of genes up‐regulated (A) and down‐regulated (B) by IF, rIIS, and combined treatments compared to the control. The top three GO terms with the lowest adjusted *p*‐values are listed, each with up to three example gene names.

**FIGURE 6:**
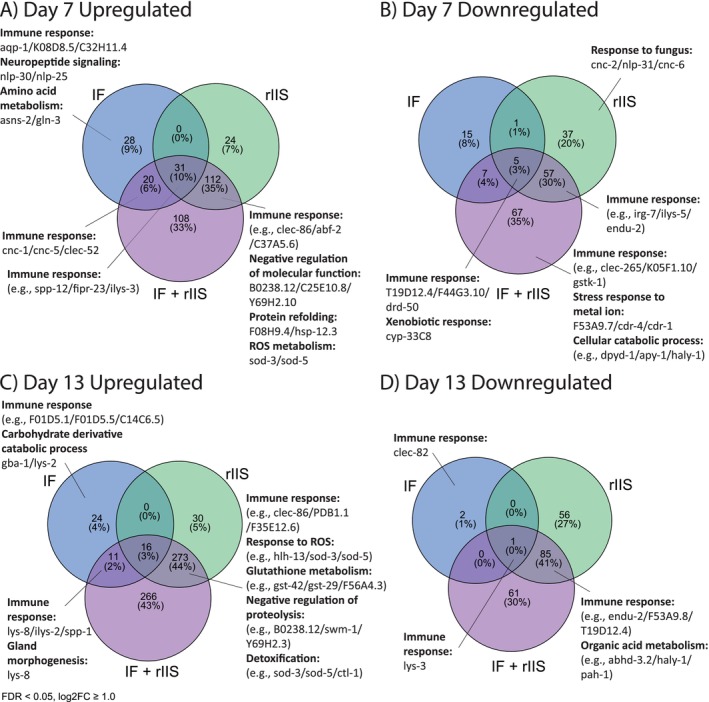
Gene Ontology analysis of differentially expressed genes across IF, rIIS and IF + rIIS treatments on day 7 and 13. GO terms showing the functions of genes up‐regulated (A) and down‐regulated (B) on day 7, and up‐regulated (C) and down‐regulated (D) on day 13 by different treatments. GO terms are ranked by adjusted *p*‐value, with the most statistically significant terms listed first. Up to three example genes are listed per GO term.

To understand the shared and unique mechanisms between IF and rIIS, we used Venn diagrams for Gene Ontology (Figures 5 and 6). On day 1, all treatments upregulated genes involved in developmental processes. Moreover, rIIS uniquely upregulated 55% of differentially expressed genes. These genes were related to processes such as DNA and RNA metabolism (Figure 5A). On the other hand, all treatments downregulated genes involved in biosynthesis, a change known to promote longevity (Hansen et al. 2007). This implies that similar mechanisms could contribute to extending reproductive‐span and lifespan across IF, rIIS, and combined treatments, particularly early in life. Still, the level of downregulation is important because decreasing the biosynthesis can be harmful to reproduction (Pan et al. 2007). In fact, we observed that IF uniquely downregulates genes involved in carbohydrate biosynthesis on day 1. Differential regulation of biosynthetic processes can be one of the reasons why rIIS had no effect on early reproduction while IF was detrimental. 56% of differentially regulated genes were uniquely downregulated by rIIS, involved in processes such as neuropeptide signalling, tricarboxylic acid cycle and energy metabolism (Figure 5B). In summary, during early adulthood, all treatments upregulated genes involved in developmental processes while uniquely influencing various metabolic and biosynthetic pathways. These indicate that both overlapping and distinct mechanisms that are regulated by IF and rIIS can be affecting ageing during early adulthood.

On day 7, IF, rIIS and their combination differentially regulated several immune genes (Figure 6A,B). While many immune‐related genes were shared between rIIS and the combined treatment, others were shared between IF and the combined treatment. Specifically, the combined and IF treatments upregulated immune genes *cnc*‐*1*, *cnc*‐*5* and *clec*‐*52*, which act through pathways beyond IIS, such as p38 MAPK pathway (Huang et al. 2020) (Figure 6A). The combined treatment also uniquely downregulated several immunity‐related genes (Figure 6B). Intriguingly, among them, *cdr*‐*4*, previously reported to be upregulated by rIIS (Weng et al. 2024), showed the opposite response in the combined treatment. These results suggest that while IF and rIIS share some immune genes, IF may influence other genes involved in pathways other than insulin signalling, highlighting the complex interaction between IF and rIIS in modulating immune function.

On day 13, similar to day 7, the combination of IF and rIIS differentially regulated various immune genes. Many were jointly regulated by rIIS and the combined treatment, with much fewer shared between IF and the combined treatment (Figure 6C,D). rIIS and the combined treatment both upregulated genes involved in several processes, including immune response, response to ROS, and amino acid metabolism, while IF and the combined treatments jointly upregulated immune genes *lys*‐*8*, *spp*‐*1* and *ilys*‐*2* (Figure 6C). Notably, *lys*‐*8* has been reported to be influenced by IIS, as well as other pathways (Alper et al. 2007). Interestingly, both *spp*‐*1* and *ilys*‐*2* have been reported to be downregulated by rIIS (Weng et al. 2024), in contrast to their upregulation by IF and the combined treatment. So, only *lys*‐*8* was regulated in the same direction as rIIS. Both rIIS and the combined treatment downregulated genes related to immune response and organic acid metabolism (Figure 6D), but no genes were jointly downregulated by IF and the combined treatment. In short, at older ages, the combination of IF and rIIS regulates a substantial number of processes similar to rIIS alone, while some processes are also influenced by IF. These results emphasise the age‐dependent effects of combining IF and rIIS, highlighting the potential role of immune function in ageing.

Finally, considering the importance of biosynthesis and immunity in our GO analyses, we investigated which differentially expressed direct DAF‐16 targets in the combined treatment were significantly enriched in one or both of these two processes. We found that, particularly early in life, several direct DAF‐16 target genes involved in suppressing biosynthesis or regulating immunity were differentially regulated (Tables S2 and S3). However, the number of direct DAF‐16 targets involved in these processes declined later in life. While direct DAF‐16 targets are a key indicator of DAF‐16 activity, many other genes are indirectly regulated by DAF‐16, for example through transcription factors regulated by DAF‐16 (Sun, Chen, and Wang 2017). So, DAF‐16's role in modulating biosynthesis and immunity is potentially broader.

## 4 Discussion

We found that adulthood‐only reduced insulin signalling (rIIS) via *daf*‐*2* knockdown slows down reproductive ageing and, in doing so, increases lifetime reproductive success in *C. elegans* hermaphrodites because early life reproduction via selfing was unaffected. This effect was particularly pronounced when animals experienced food shortage in the form of IF. By examining the nuclear localisation of DAF‐16 across different ages, we showed that the combined effects of early‐adulthood IF and rIIS led to higher total nuclear localisation of this transcription factor, potentially maximising its activity. Specifically, DAF‐16 localisation in the combined treatment was mainly driven by IF instead of rIIS in early life, while the opposite was true later in life. Genome‐wide RNA sequencing showed that IF and rIIS shape gene expression in an age‐dependent manner. The GO terms analyses suggest that genes involved in biosynthesis and immunity are potential candidates for explaining the additive effects of IF and rIIS on ageing. The reduction in biosynthesis during early adulthood at least partially aligns with the DST, suggesting a trade‐off between growth and maintenance (resource allocation). In contrast, the regulation of immunity genes later in life is broadly in line with the DTA, which posits that ageing results from suboptimal gene expression in late life due to the weakening of natural selection on biological function in adulthood.

Previous work shows a complex picture with respect to the suggested role of insulin signalling in reproduction in *C. elegans* (Lee and Lee 2022). Different studies reported that while some *daf*‐*2* alleles can result in sterility, others lead to the same or extended reproductive lifespan of selfing hermaphrodites (Hughes et al. 2007). However, self‐fertile *C. elegans* hermaphrodites are sperm limited (around 300 sperm), and mating can substantially increase late reproduction and lead to higher lifetime reproductive success. Therefore, while some reproductive ageing in *C. elegans* hermaphrodites occurs prior to cessation of sperm, it is key to study reproductive ageing in mated hermaphrodites that have unlimited access to sperm while still producing oocytes. Mated *daf*‐*2* (*e1370*) hermaphrodites can have increased reproductive lifespan but reduced rate of reproduction and low lifetime reproductive success compared to wild‐type animals (Hughes et al. 2007). Later work showed that *daf*‐*2* mutants have improved maintenance of oocyte quality with age (Luo et al. 2010). Because early‐life reproduction is more important for fitness than late‐life reproduction, given *C. elegans* ' life history (Chen et al. 2006), this suggests a genetic trade‐off or constraint that links reproductive lifespan extension to reduced fitness. It is important to emphasise that such a trade‐off is unlikely to be resource‐dependent because neither reduction nor increase in early‐life offspring production directly affects reproductive ageing (Hughes et al. 2007; Luo et al. 2009, 2010). Finally, later work suggested that mated *daf*‐*2* (*e1370*) hermaphrodites may actually suffer from reduced reproductive lifespan and reduced overall reproduction (Mendenhall et al. 2011). The discrepancy between different studies likely results from the timing of mating during life course and differences in animal husbandry, but it is clear that lifelong reduction in IIS in *daf*‐*2* mutants is likely to carry substantial fitness costs, even if it has positive effects on reproductive ageing under certain conditions.

While we found that adulthood‐only *daf*‐*2* RNAi improves late reproduction without negative effects on the early self‐fertile stage together with increased longevity, it remains possible that there is a trade‐off between slower reproductive ageing and offspring quality. We have previously shown that adulthood‐only *daf*‐*2* RNAi improves offspring quality of self‐fertile hermaphrodites in a benign environment (Lind et al. 2019), has no effect on offspring quality in fluctuating stressful environments (Carlsson et al. 2021), and has overall positive effects on fitness and lineage survival in a long‐term multigenerational scenario (Duxbury et al. 2022). Here, we found that offspring produced late in life by *daf*‐*2* RNAi mated hermaphrodites had similar lifetime reproductive success and survival compared to wild‐type worms. Overall, these results suggest that *daf*‐*2* RNAi‐mediated improvement in reproductive ageing is both general across benign and stressful environments and does not trade‐off with offspring quality.

Interestingly, we found that IF during early‐adulthood, but not late‐adulthood, increases survival. This result aligns with previous work in *D. melanogaster* fruit flies, suggesting that IF has a greater impact on survival when applied early, but not late, in life (Catterson et al. 2018; Ulgherait et al. 2021). In fruit flies, 5 days fasting‐2 days eating IF regimen increased lifespan when applied during early or mid‐adulthood. However, when applied during late‐adulthood, it shortened lifespan (Catterson et al. 2018). Similarly, 18 h fasting‐6 h eating IF regimen also increased lifespan in fruit flies when applied during early, but not late, adulthood (Ulgherait et al. 2021). Our findings in worms further support the importance of the timing of the treatment, with early‐adulthood being more beneficial.

Crucially, early‐adulthood IF further increased survival in the presence of rIIS in all three strains, suggesting that IF may be working at least in part through a different route than IIS. However, when applied alone, early‐adulthood IF had varying effects on different strains. As these strains originated from different parts of the world (the UK, USA and Portugal), their environmental backgrounds may have shaped their responses to IF differently (Barriere and Félix 2005). Although early or late‐adulthood IF was not previously compared in *C. elegans* , it was shown that applying IF throughout the entire lifespan increases longevity (Honjoh et al. 2009; Uno et al. 2013). In these studies, a 2‐day fasting‐2‐day eating regimen increased survival in *C. elegans* through an IIS‐dependent mechanism, contrasting with our findings (Honjoh et al. 2009; Uno et al. 2013). Our IF regimen differs from previous studies, which used a 2‐day IF and eating cycle (Uno et al. 2013), whereas in our experiment, we applied 9 h of fasting on alternate days. We chose this IF regimen because previous research found that in *C. elegans* , a significant portion of ageing‐related genes is upregulated between 6 and 9 h of fasting (Uno et al. 2013). Our IF regimen is perhaps more similar to the time‐restricted feeding regimen used in fruit flies, which was suggested to work independently of inhibition of insulin‐like signalling (Ulgherait et al. 2021).

To better understand the mechanisms underlying our results, we first explored DAF‐16 nuclear localisation. DAF‐16 is a key transcription factor which acts downstream of nutrient‐sensing signalling and germline signalling pathways (Sun, Chen, and Wang 2017). As a response to signals related to food availability or germline maintenance, it enters the nucleus and transcriptionally activates pro‐longevity genes (Sun, Chen, and Wang 2017). In our experiment, DAF‐16 remained in the cytoplasm in the presence of food and when the IIS pathway was active (control) during early and later adulthood (consistent with [Weinkove et al. 2006], but see [Li et al. 2019]). However, when food is scarce (IF treatment), when the IIS is inactivated (rIIS treatment), or both (combined treatment), DAF‐16 is expected to relocate to the nucleus (Henderson and Johnson 2001). Our findings indicate that in early‐adulthood, fasting causes DAF‐16 to enter the nucleus more than rIIS. This can be due to IF activating pathways other than IIS (Ulgherait et al. 2021) and/or because IIS is not sufficiently knocked down by RNAi compared to fasting (Venz et al. 2021). Conversely, fasting is no longer that effective in later adulthood, while rIIS worms maintain the same DAF‐16 nuclear localisation as observed in their youth. DAF‐16 has been shown to rapidly respond to changing food availability by moving into the nucleus shortly after fasting initiation and returning to the cytoplasm upon resuming a normal diet (Henderson and Johnson 2001). Hence, in our IF treatment, DAF‐16 is expected to be in the nucleus only during fasting periods (9 h on alternate days) and back in the cytoplasm during non‐fasting periods. In the rIIS treatment, DAF‐16 is expected to be in the nucleus constantly, at least during the first week of lifespan, because IIS is constantly reduced by RNAi treatment. In fact, deactivating the IIS pathway has been shown to cause DAF‐16 to stay in the nucleus even until later ages, as late as day 11 (Weinkove et al. 2006). In the combined treatment, worms experience both intermittent nuclear localisation due to IF and the constant nuclear localisation due to rIIS, and benefit from both treatments. These findings support the hypothesis that IF and rIIS are modulating DAF‐16 localisation in an age‐dependent manner, leading to potentially increased DAF‐16 activity in combined treatment. This is further supported by the fact that some direct DAF‐16 targets are only expressed in the combined treatment at later ages.

Our gene expression analyses have revealed potential mechanisms that could play a critical role in improving late‐life reproduction as well as increasing longevity. While we highlight biosynthesis and immunity as key processes in modulating reproductive ageing, previous studies explored their effects mainly in lifespan extension (Hansen et al. 2007; Kurz and Tan 2004). For example, we have previously shown that suppressing protein biosynthesis during adulthood improves survival and egg size (Lind et al. 2021). How these processes affect reproductive ageing remains less explored. Moreover, other processes can also hold significant potential for reproductive ageing and longevity. For example, we found that *sucl*‐*2* (involved in energy metabolism) is downregulated by IF + rIIS during early adulthood. In line with this, a recent study shows that knocking down *sucl*‐*2* increases reproductive lifespan (Lee et al. 2023).

Our study suggests that reduced insulin signalling in adulthood ameliorates reproductive ageing in benign and food‐limited environments without costs to reproduction or offspring quality. Notably, the benefits of adulthood‐only rIIS to late‐life reproduction and survival becomes more apparent when animals experience fluctuating food shortages in the form of IF. Specifically, we observed that early‐adulthood IF and adulthood‐only rIIS additively enhance both survival and late‐life reproduction. The dynamic regulation of DAF‐16 nuclear localisation in an age‐specific manner suggests a finely tuned mechanism through which IF and rIIS exert their effects. Our exploration of age‐specific gene expression patterns suggests that both shared and unique regulatory pathways may contribute to the additive effects of IF and rIIS on life‐history traits. These results align with the hypothesis that reduction in the force of natural selection after reproductive maturity results in the evolution of suboptimal gene expression in adulthood, causing ageing (de Magalhães and Church 2005; Lemaître et al. 2024).

Nevertheless, the downregulation of certain genes involved in immunity by the combined treatment could be detrimental and pose challenges in the presence of pathogens. For example, we found that *lys*‐*3*, a lysozyme induced in response to pathogenic bacteria (Boehnisch et al. 2011), is consistently downregulated in all three treatments during late adulthood, despite evidence that its overexpression can extend lifespan in *C. elegans* (Gallotta et al. 2020).

It has been previously suggested that both DTA and DST‐like processes can play a role in the evolution of ageing and that these processes are not mutually exclusive (Maklakov and Chapman 2019; Lemaître et al. 2024). The additive increase in lifespan and late‐life reproduction, when rIIS was combined with IF, aligns with this suggestion because IF directly limits reproduction, and combining rIIS with IF to further slow down reproductive ageing comes at the cost of early‐life reproduction. Moreover, both down‐ and up‐regulation of certain immune genes by rIIS and IF indicates that slowing down ageing can be costly in less benign environments, especially when immune challenges are present (but see [Carlsson et al. 2021]). Further research is necessary to establish the relative contributions of resource allocation trade‐offs and suboptimal age‐specific gene expression to the evolution and expression of ageing across a broad range of taxa. The lack of functional validation of specific genes in our study is a limitation, as such analyses could clarify these contributions. Addressing this in future studies will further strengthen the mechanistic understanding of ageing and its modulation through IF and rIIS.

## Author Contributions

Conceptualization: Z.S., A.S., K.H., A.A. and A.A.M. methodology: Z.S., K.H., H.C., Z.C. and D.C. data analysis: Z.S. and A.S. writing – original draft: Z.S. and A.A.M. writing – review and editing: Z.S., A.A.M., A.A., A.S., K.H., H.C., Z.C. and D.C.

## Conflicts of Interest

The Authors declare no conflicts of interest.

## Supporting information


**Data S1.** Sultanova_data.

Data S2.

## Data Availability

All raw data related to RNA sequencing is deposited at the European Nucleotide Archive (https://www.ebi.ac.uk/ena/browser/home) under the accession number PRJEB80737. The remaining data are available in the Supporting Information.
