# Supplementary material for: Optimising Age‐Specific Insulin Signalling to Slow Down Reproductive Ageing Increases Fitness in Different Nutritional Environments
Source: Aging Cell. 2025 Jan 24;24(5):e14481. doi: 10.1111/acel.14481 (PMC12073930; doi:10.1111/acel.14481)
Supplement: Supplementary file 2 — Data S2. [file ACEL-24-e14481-s002.docx]

# Supplementary Materials

| **IF exposure** | **Matricide** | **Strain** | **Treatment** | **Sample Size** | **Mean Lifespan** | **SD of Mean Lifespan** | **Median Lifespan** | **Maximum Lifespan** |
| --- | --- | --- | --- | --- | --- | --- | --- | --- |
| early-life | included | N2 | Control | 100 | 17.69 | 3.80 | 18 | 25 |
| early-life | included | N2 | IF | 100 | 19.82 | 5.97 | 20 | 32 |
| early-life | included | N2 | rIIS | 100 | 34.48 | 9.18 | 36 | 50 |
| early-life | included | N2 | IF + rIIS | 100 | 37.67 | 12.37 | 41 | 57 |
| early-life | included | P | Control | 100 | 17.56 | 6.20 | 19 | 31 |
| early-life | included | P | IF | 100 | 15.51 | 7.69 | 16 | 28 |
| early-life | included | P | rIIS | 100 | 23.10 | 9.87 | 26 | 42 |
| early-life | included | P | IF + rIIS | 100 | 23.00 | 14.26 | 24 | 45 |
| early-life | included | U | Control | 100 | 19.96 | 7.36 | 22.5 | 30 |
| early-life | included | U | IF | 100 | 20.91 | 10.49 | 25 | 37 |
| early-life | included | U | rIIS | 100 | 27.60 | 10.37 | 31 | 45 |
| early-life | included | U | IF + rIIS | 100 | 30.93 | 15.38 | 35 | 56 |
| early-life | not included | N2 | Control | 96 | 18.16 | 3.10 | 18.5 | 25 |
| early-life | not included | N2 | IF | 98 | 20.37 | 5.18 | 20 | 32 |
| early-life | not included | N2 | rIIS | 99 | 34.83 | 8.58 | 36.5 | 50 |
| early-life | not included | N2 | IF + rIIS | 97 | 39.43 | 11.43 | 41 | 60 |
| early-life | not included | P | Control | 92 | 18.62 | 5.16 | 19 | 31 |
| early-life | not included | P | IF | 96 | 16.61 | 7.14 | 18 | 28 |
| early-life | not included | P | rIIS | 85 | 26.64 | 6.53 | 27 | 42 |
| early-life | not included | P | IF + rIIS | 94 | 26.77 | 12.37 | 29 | 45 |
| early-life | not included | U | Control | 87 | 22.20 | 5.11 | 23 | 30 |
| early-life | not included | U | IF | 94 | 23.48 | 8.67 | 26 | 37 |
| early-life | not included | U | rIIS | 92 | 29.99 | 7.70 | 32 | 45 |
| early-life | not included | U | IF + rIIS | 94 | 34.90 | 12.22 | 36 | 56 |
| late-life | included | N2 | Control | 100 | 16.62 | 5.25 | 16 | 27 |
| late-life | included | N2 | IF | 100 | 16.17 | 4.59 | 16 | 25 |
| late-life | included | N2 | rIIS | 100 | 28.25 | 7.17 | 29 | 46 |
| late-life | included | N2 | IF + rIIS | 100 | 28.14 | 9.49 | 30 | 48 |
| late-life | not included | N2 | Control | 95 | 17.14 | 4.84 | 18 | 27 |
| late-life | not included | N2 | IF | 99 | 16.31 | 4.40 | 16 | 25 |
| late-life | not included | N2 | rIIS | 97 | 28.99 | 5.95 | 29 | 46 |
| late-life | not included | N2 | IF + rIIS | 93 | 29.89 | 7.38 | 30.5 | 48 |
| Inter-gen | included | N2 | Control | 58 | 14.58 | 4.48 | 17 | 22 |
| Inter-gen | included | N2 | IF | 76 | 15.11 | 4.69 | 17 | 23 |
| Inter-gen | included | N2 | rIIS | 37 | 15.19 | 4.65 | 17 | 22 |
| Inter-gen | included | N2 | IF + rIIS | 58 | 16.16 | 4.92 | 17 | 24 |
| Inter-gen | not included | N2 | Control | 55 | 15.20 | 3.69 | 17 | 22 |
| Inter-gen | not included | N2 | IF | 71 | 15.94 | 3.59 | 17 | 23 |
| Inter-gen | not included | N2 | rIIS | 36 | 15.54 | 4.22 | 17 | 22 |
| Inter-gen | not included | N2 | IF + rIIS | 54 | 17.11 | 3.54 | 17.5 | 24 |

**Table S1.** Descriptive survival statistics corresponding to different treatments and worm strains.

| **Gene ID** | **Gene name** | **Function** | **Regulation** | **Day** |
| --- | --- | --- | --- | --- |
| WBGene00018846 | eef-1B.1 | Amide/Peptide biosynthesis | down | 1 |
| WBGene00003026 | lin-41 | Amide/Peptide biosynthesis | down | 1 |
| WBGene00004241 | puf-5 | Amide/Peptide biosynthesis | down | 1 |
| WBGene00004435 | rpl-23 | Amide/Peptide biosynthesis | down | 1 |
| WBGene00004452 | rpl-38 | Amide/Peptide biosynthesis | down | 1 |
| WBGene00004492 | rps-23 | Amide/Peptide biosynthesis | down | 1 |
| WBGene00012015 | T25B9.9 | Immune response | down | 1 |
| WBGene00001749 | gst-1 | Immune response | down | 1 |
| WBGene00022538 | ZC190.4 | Negative regulation of biosynthesis | up | 1 |
| WBGene00015547 | ain-1 | Negative regulation of biosynthesis | up | 1 |
| WBGene00000414 | cec-1 | Negative regulation of biosynthesis | up | 1 |
| WBGene00001208 | egl-44 | Negative regulation of biosynthesis | up | 1 |
| WBGene00017699 | flcn-1 | Negative regulation of biosynthesis | up | 1 |
| WBGene00001972 | hmg-1.2 | Positive regulation of biosynthesis | up | 1 |
| WBGene00002147 | ire-1 | Positive regulation of biosynthesis | up | 1 |
| WBGene00002717 | let-526 | Positive regulation of biosynthesis | up | 1 |
| WBGene00003003 | lin-14 | Negative regulation of biosynthesis | up | 1 |
| WBGene00011259 | miz-1 | Positive regulation of biosynthesis | up | 1 |
| WBGene00011280 | pelo-1 | Negative regulation of biosynthesis | up | 1 |
| WBGene00004773 | sem-4 | Negative regulation of biosynthesis | up | 1 |
| WBGene00004862 | sma-9 | Negative regulation of biosynthesis | up | 1 |
| WBGene00006853 | unc-130 | Negative regulation of biosynthesis | up | 1 |
| WBGene00006779 | unc-43 | Positive regulation of biosynthesis | up | 1 |
| WBGene00000102 | akt-1 | Immune response | up | 1 |
| WBGene00000103 | akt-2 | Immune response | up | 1 |
| WBGene00001196 | egl-30 | Immune response | up | 1 |
| WBGene00001398 | fat-6 | Immune response | up | 1 |
| WBGene00003476 | mtm-3 | Immune response | up | 1 |
| WBGene00007918 | sphk-1 | Immune response | up | 1 |
| WBGene00006923 | vhp-1 | Immune response | up | 1 |
| WBGene00000914 | daf-19 | Immune response  Positive regulation of biosynthesis | up | 1 |
| WBGene00001441 | fkh-9 | Immune response  Positive regulation of biosynthesis | up | 1 |
| WBGene00010700 | nipi-3 | Immune response  Negative regulation of biosynthesis | up | 1 |
| WBGene00010251 | sta-2 | Immune response  Positive regulation of biosynthesis | up | 1 |
| WBGene00011232 | pck-2 | Alcohol/Carbohydrate biosynthesis | up | 13 |

**Table S2.** Biosynthesis and immunity related direct DAF-16 targets that were differentially regulated by the combination of IF and rIIS on different days of lifespan. FDR< 0.05 & log2FC ≥ 1.0 (main analysis).

| **Gene ID** | **Gene name** | **Function** | **Regulation** | **Day** |
| --- | --- | --- | --- | --- |
| WBGene00008948 | F19B6.1 | Organophosphate biosynthesis | down | 1 |
| WBGene00018519 | F46H5.3 | Organophosphate biosynthesis | down | 1 |
| WBGene00009219 | dpm-3 | Amide/Peptide biosynthesis | down | 1 |
| WBGene00018846 | eef-1B.1 | Amide/Peptide biosynthesis | down | 1 |
| WBGene00001227 | eif-3.D | Organic acid biosynthesis | down | 1 |
| WBGene00001239 | elo-1 | Amide/Peptide biosynthesis | down | 1 |
| WBGene00003026 | lin-41 | ATP synthesis | down | 1 |
| WBGene00006463 | nduf-2.2 | Amide/Peptide biosynthesis | down | 1 |
| WBGene00004241 | puf-5 | Amide/Peptide biosynthesis | down | 1 |
| WBGene00004435 | rpl-23 | Amide/Peptide biosynthesis | down | 1 |
| WBGene00004452 | rpl-38 | Amide/Peptide biosynthesis | down | 1 |
| WBGene00004492 | rps-23 | Organic acid biosynthesis | down | 1 |
| WBGene00005022 | sqv-4 | Organophosphate biosynthesis | down | 1 |
| WBGene00018984 | F56F10.1 | Immune response | down | 1 |
| WBGene00012015 | T25B9.9 | Immune response | down | 1 |
| WBGene00000423 | ced-9 | Immune response | down | 1 |
| WBGene00001749 | gst-1 | Immune response | down | 1 |
| WBGene00022538 | ZC190.4 | Negative regulation of biosynthesis | up | 1 |
| WBGene00015547 | ain-1 | Negative regulation of biosynthesis | up | 1 |
| WBGene00000414 | cec-1 | Negative regulation of biosynthesis | up | 1 |
| WBGene00001208 | egl-44 | Negative regulation of biosynthesis | up | 1 |
| WBGene00017699 | flcn-1 | Negative regulation of biosynthesis | up | 1 |
| WBGene00001851 | hif-1 | Positive regulation of biosynthesis | up | 1 |
| WBGene00001972 | hmg-1.2 | Positive regulation of biosynthesis | up | 1 |
| WBGene00002147 | ire-1 | Positive regulation of biosynthesis | up | 1 |
| WBGene00002717 | let-526 | Positive regulation of biosynthesis | up | 1 |
| WBGene00003003 | lin-14 | Negative regulation of biosynthesis | up | 1 |
| WBGene00011259 | miz-1 | Positive regulation of biosynthesis | up | 1 |
| WBGene00003638 | nhr-48 | Negative regulation of biosynthesis | up | 1 |
| WBGene00011280 | pelo-1 | Negative regulation of biosynthesis | up | 1 |
| WBGene00004773 | sem-4 | Negative regulation of biosynthesis | up | 1 |
| WBGene00004862 | sma-9 | Negative regulation of biosynthesis | up | 1 |
| WBGene00006853 | unc-130 | Negative regulation of biosynthesis | up | 1 |
| WBGene00006779 | unc-43 | Positive regulation of biosynthesis | up | 1 |
| WBGene00000102 | akt-1 | Immune response | up | 1 |
| WBGene00000103 | akt-2 | Immune response | up | 1 |
| WBGene00001196 | egl-30 | Immune response | up | 1 |
| WBGene00001398 | fat-6 | Immune response | up | 1 |
| WBGene00003476 | mtm-3 | regulation of phagocytosis | up | 1 |
| WBGene00007918 | sphk-1 | regulation of phagocytosis | up | 1 |
| WBGene00006739 | ulp-4 | Immune response | up | 1 |
| WBGene00006923 | vhp-1 | Immune response | up | 1 |
| WBGene00000914 | daf-19 | Positive regulation of biosynthesis | up | 1 |
| WBGene00001441 | fkh-9 | Positive regulation of biosynthesis | up | 1 |
| WBGene00010700 | nipi-3 | Negative regulation of biosynthesis | up | 1 |
| WBGene00004758 | sek-1 | Positive regulation of biosynthesis | up | 1 |
| WBGene00010251 | sta-2 | Positive regulation of biosynthesis | up | 1 |
| WBGene00001149 | bcat-1 | Amino acid biosynthesis | down | 13 |
| WBGene00011232 | pck-2 | Carbohydrate/lipid biosynthesis | up | 13 |
| WBGene00001441 | fkh-9 | Immune response | up | 13 |

**Table S3.** Biosynthesis and immunity related direct DAF-16 targets that were differentially regulated by the combination of IF and rIIS on different days of lifespan. FDR< 0.05 & log2FC ≥ 0.5 (relaxed threshold).


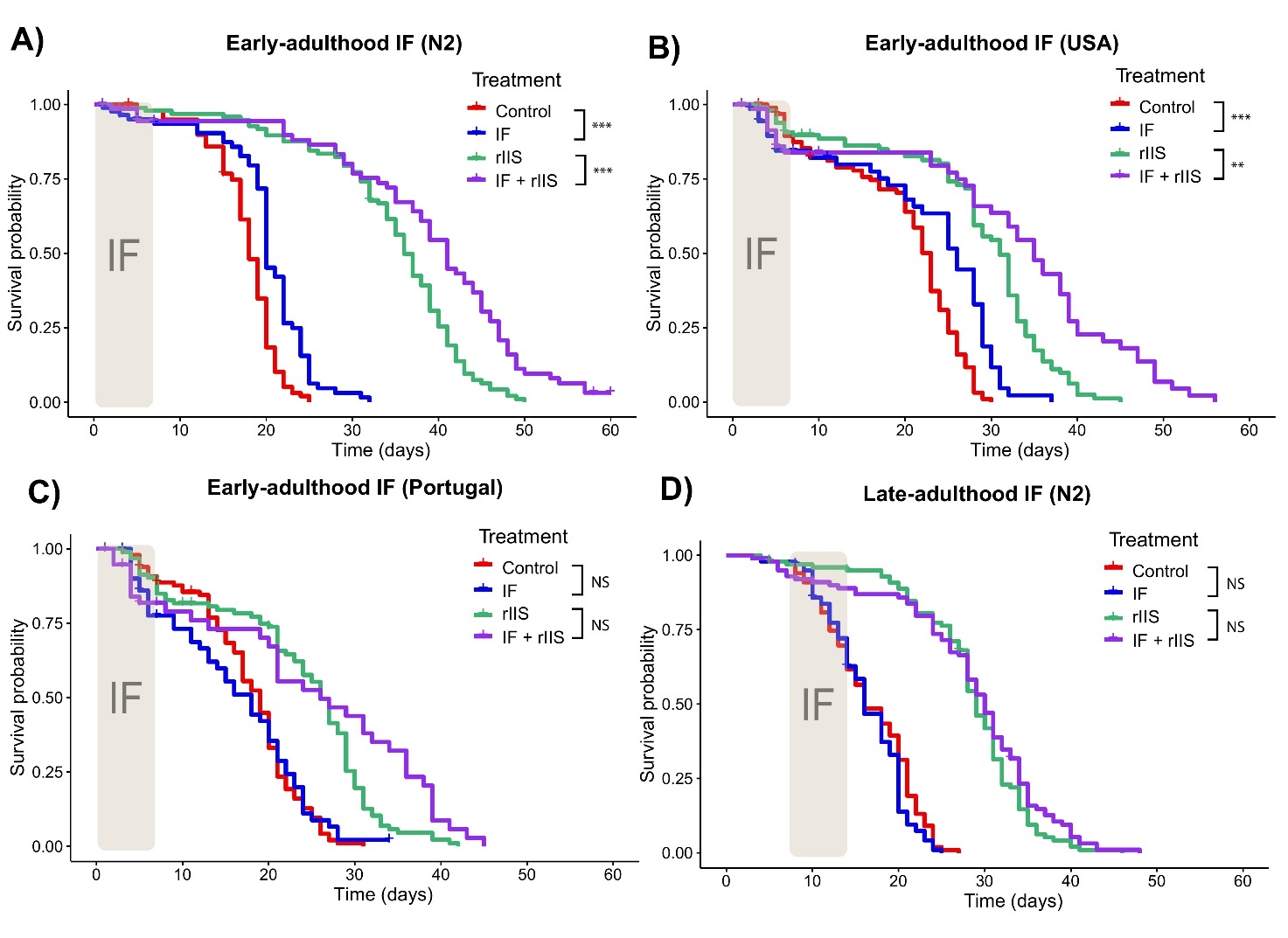


**Fig. S1.** Effect of IF and rIIS on survival including matricides. Effect of early-adulthood IF with and without rIIS on survival of N2, USA and Portugal worms (A, B and C respectively). Effect of late-adulthood IF with and without rIIS on survival of N2 (D). Significance in the divergence of survival curves was assessed using log-rank tests, with statistical significance represented as follows: NS indicates p > 0.05, * indicates p ≤ 0.05, ** indicates p ≤ 0.01, and *** indicates p ≤ 0.001.


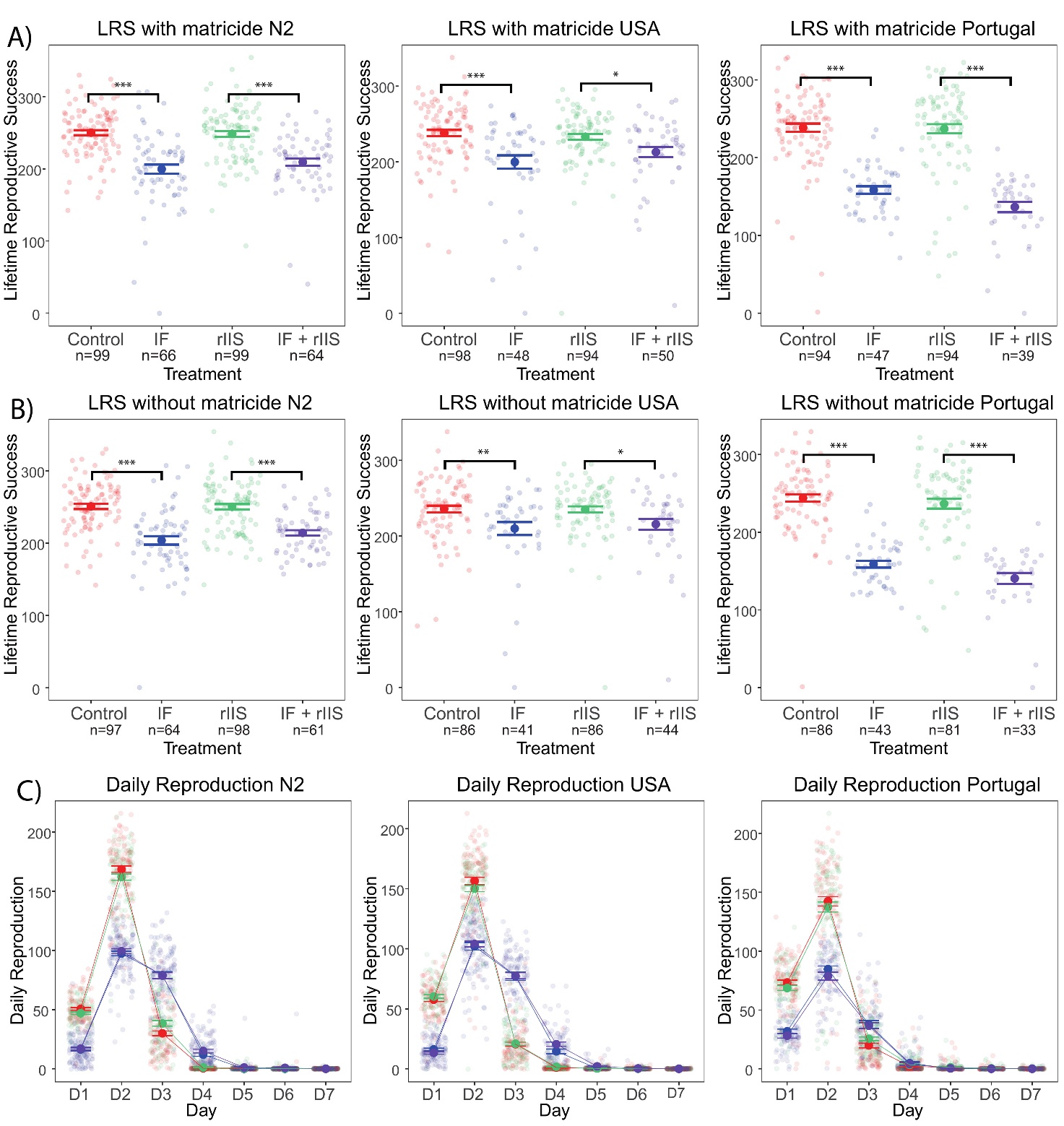


**Fig. S2.** Effect of IF and rIIS on early-life reproduction. Early-life reproduction including matricide across three different populations (A), early-life reproduction without matricide across three different populations (B), daily reproduction across three different populations (C). Differences between treatments were assessed by Tukey's HSD test with Benforini-Hochberg correction for multiple testing. NS indicates p > 0.05, * indicates p ≤ 0.05, ** indicates p ≤ 0.01, and *** indicates p ≤ 0.001.


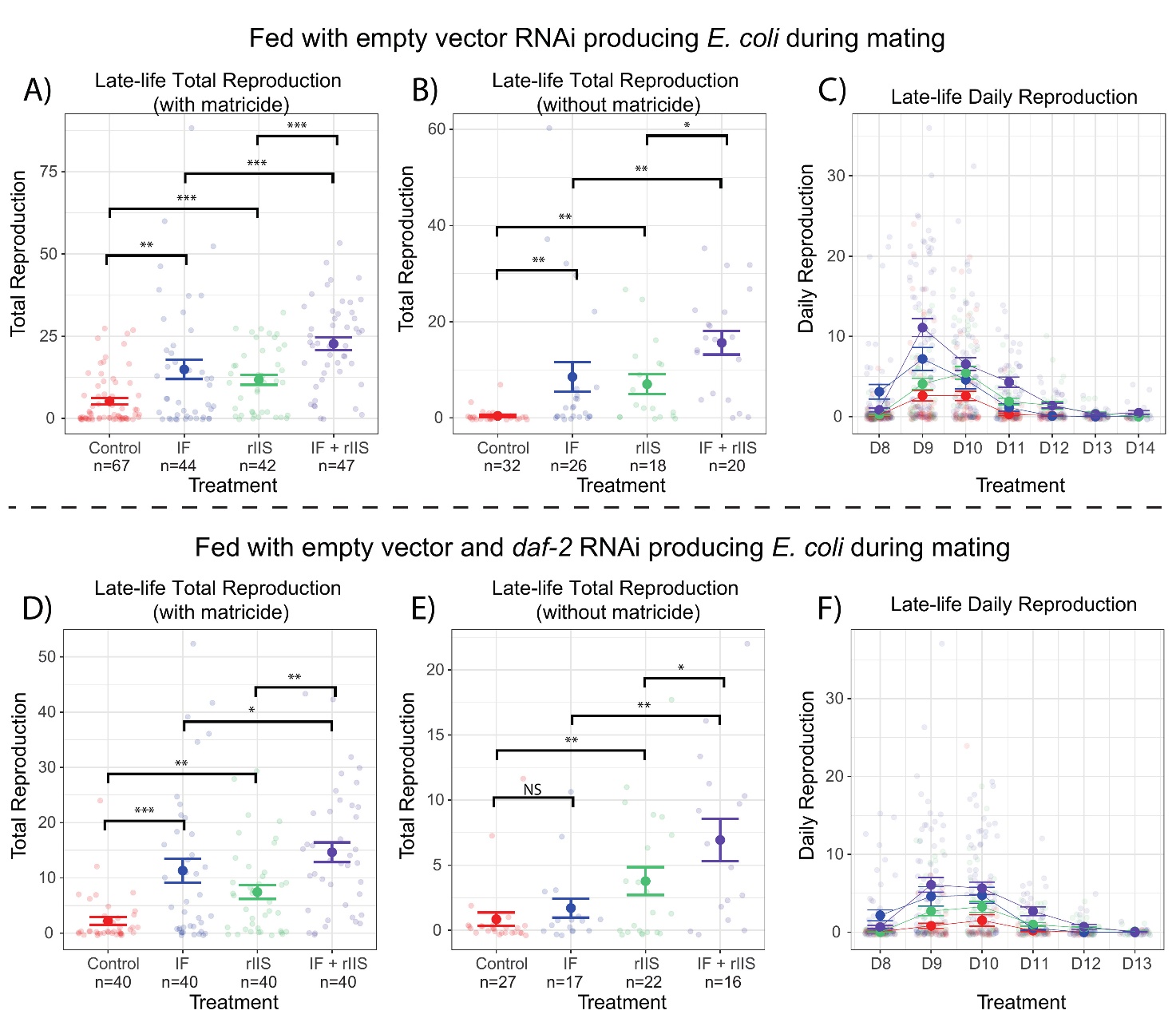


**Fig. S3.** Effect of IF and rIIS on late-life reproduction. Late-life reproduction including matricide (A), late-life reproduction without matricide (B), daily reproduction (C) in worms fed with empty vector RNAi producing *E. coli* during mating. Late-life reproduction including matricide (D), late-life reproduction without matricide (E), daily reproduction (F) in worms fed with empty vector and *daf-2* RNAi producing *E. coli* during mating. Differences between treatments were assessed by Dunn test with Benforini-Hochberg correction for multiple testing. NS indicates p > 0.05, * indicates p ≤ 0.05, ** indicates p ≤ 0.01, and *** indicates p ≤ 0.001.


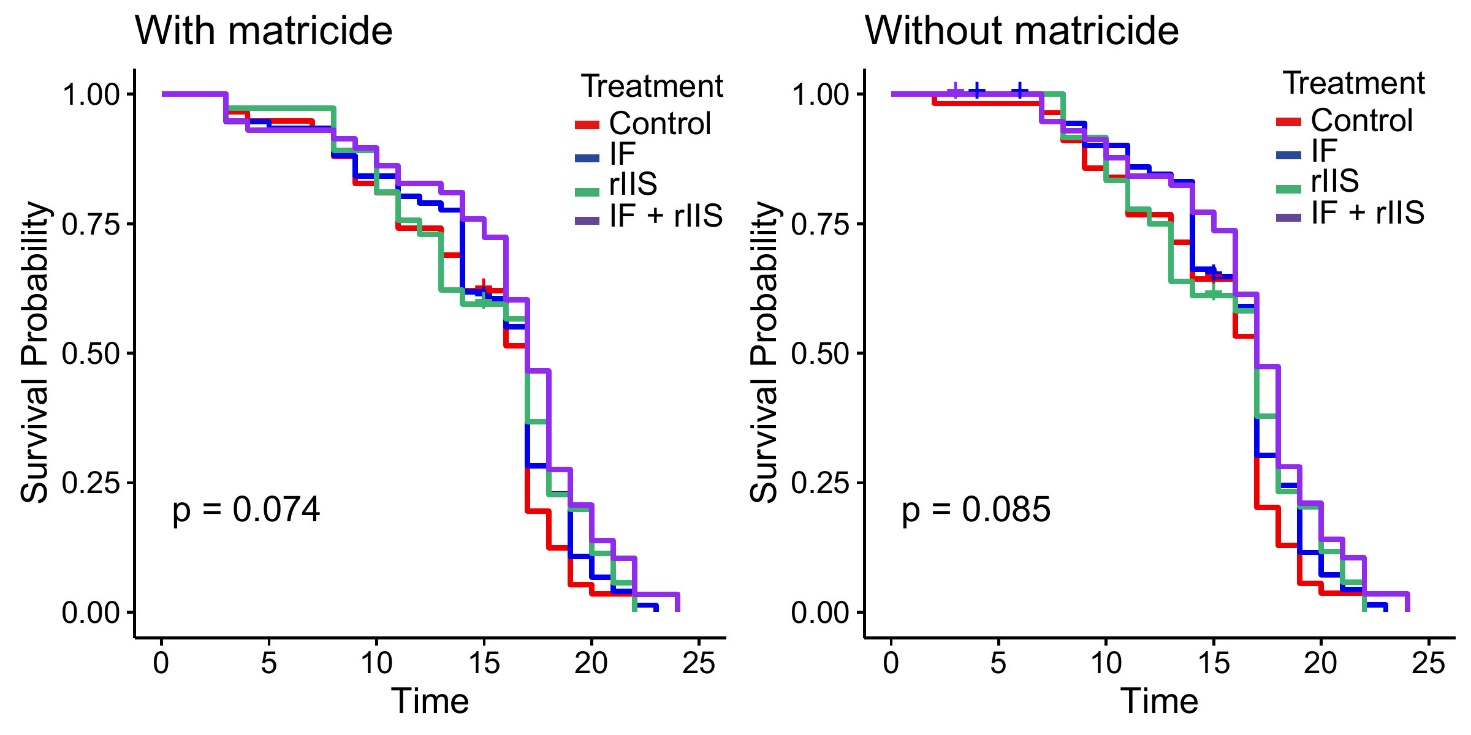


**Fig. S4.** Effect of IF and rIIS on offspring survival. Survival of offspring from parents with Control, IF, rIIS and IF + rIIS treatments. Survival with matricide (A) and without matricide (B). Significance in the divergence of survival curves was assessed using log-rank tests, with statistical significance represented as follows: NS indicates P > 0.05, * indicates P ≤ 0.05, ** indicates P ≤ 0.01, and *** indicates P ≤ 0.001.


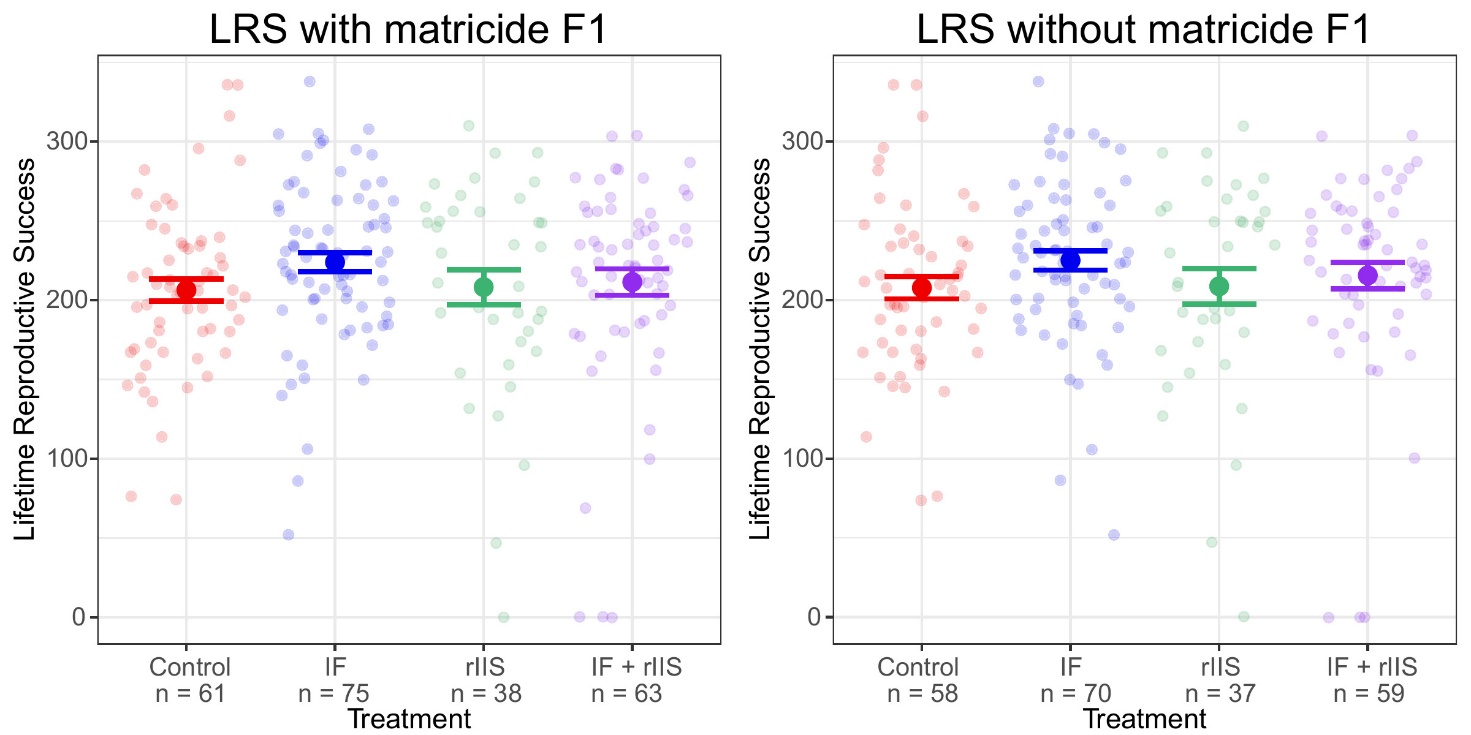


**Fig. S5.** Effect of IF and rIIS on offspring lifetime reproductive success (LRS). Lifetime reproductive success of offspring from parents with Control, IF, rIIS and IF + rIIS treatments. LRS with matricide (A), LRS without matricide (B).


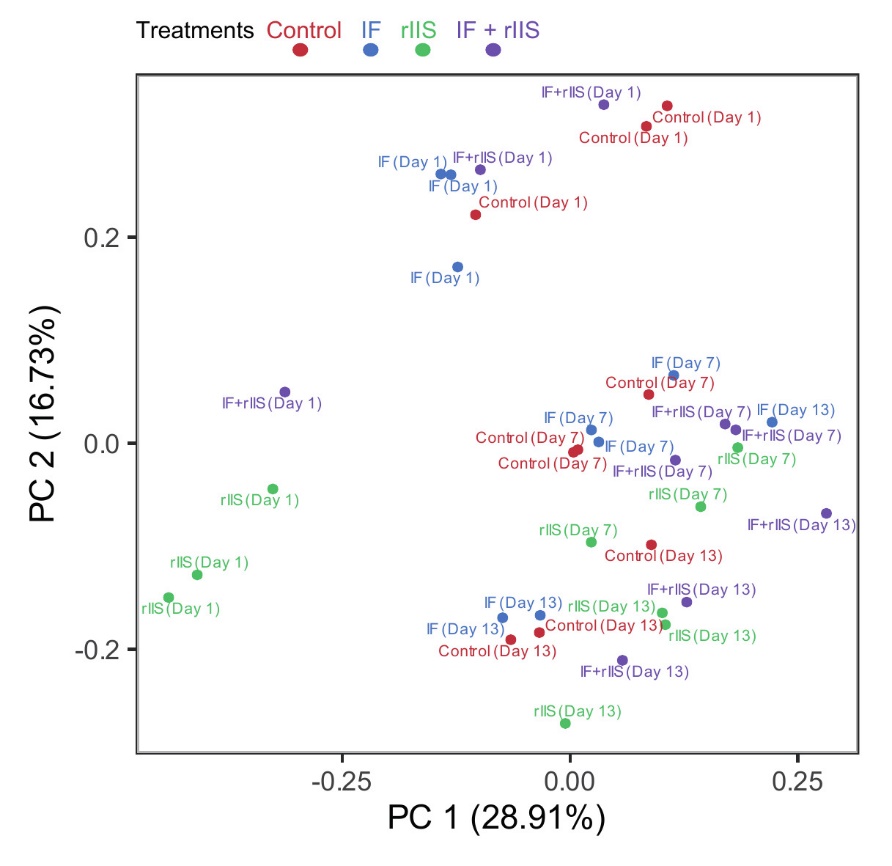


**Fig. S6.** Principal Component Analysis (PCA) of RNA-seq Samples (PC1 and PC2). PCA plots illustrating the gene expression profiles for samples under four different treatments: Control, IF, rIIS, and Combined (IF + rIIS). The plots compare the gene expression patterns at three distinct time points – days 1, 7, and 13 – to demonstrate how the treatments influence genetic activity over time.


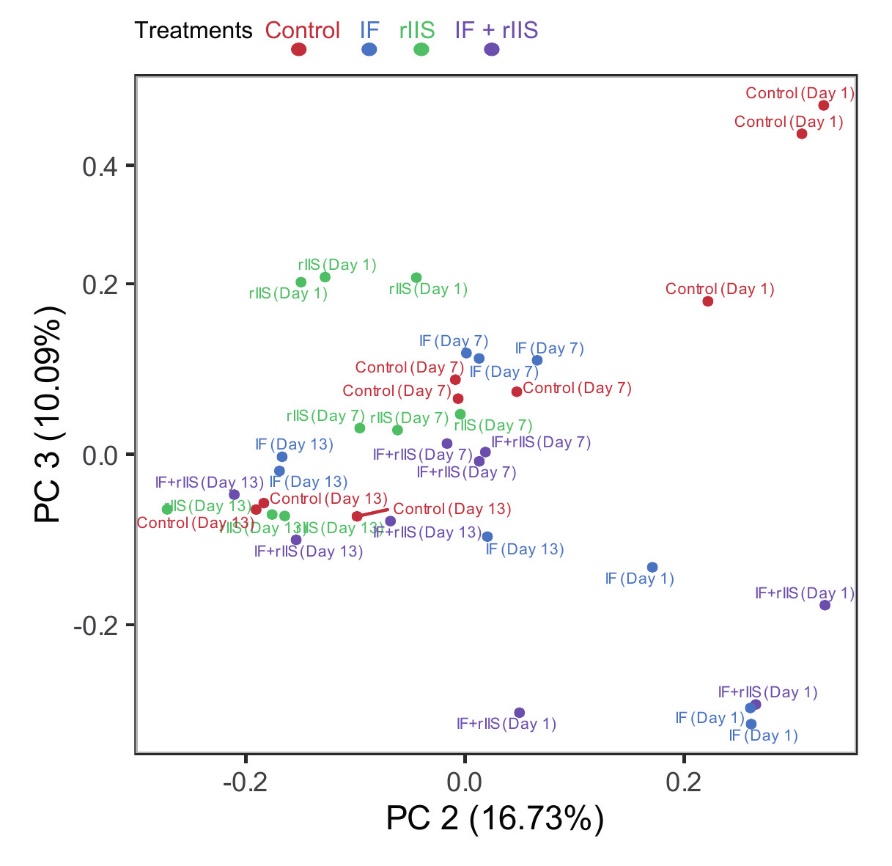


**Fig. S7.** Principal Component Analysis (PCA) of RNA-seq Samples (PC2 and PC3). PCA plots illustrating the gene expression profiles for samples under four different treatments: Control, IF, rIIS, and Combined (IF + rIIS). The plots compare the gene expression patterns at three distinct time points – days 1, 7, and 13 – to demonstrate how the treatments influence genetic activity over time.


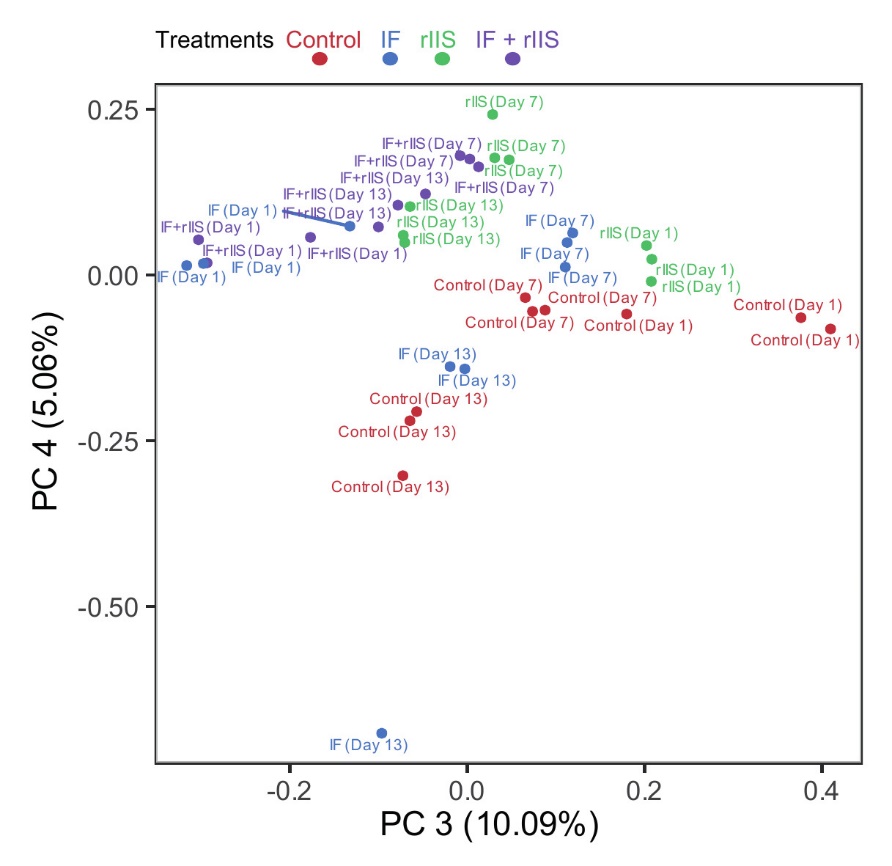


**Fig. S8.** Principal Component Analysis (PCA) of RNA-seq Samples (PC3 and PC4). PCA plots illustrating the gene expression profiles for samples under four different treatments: Control, IF, rIIS, and Combined (IF + rIIS). The plots compare the gene expression patterns at three distinct time points – days 1, 7, and 13 – to demonstrate how the treatments influence genetic activity over time.


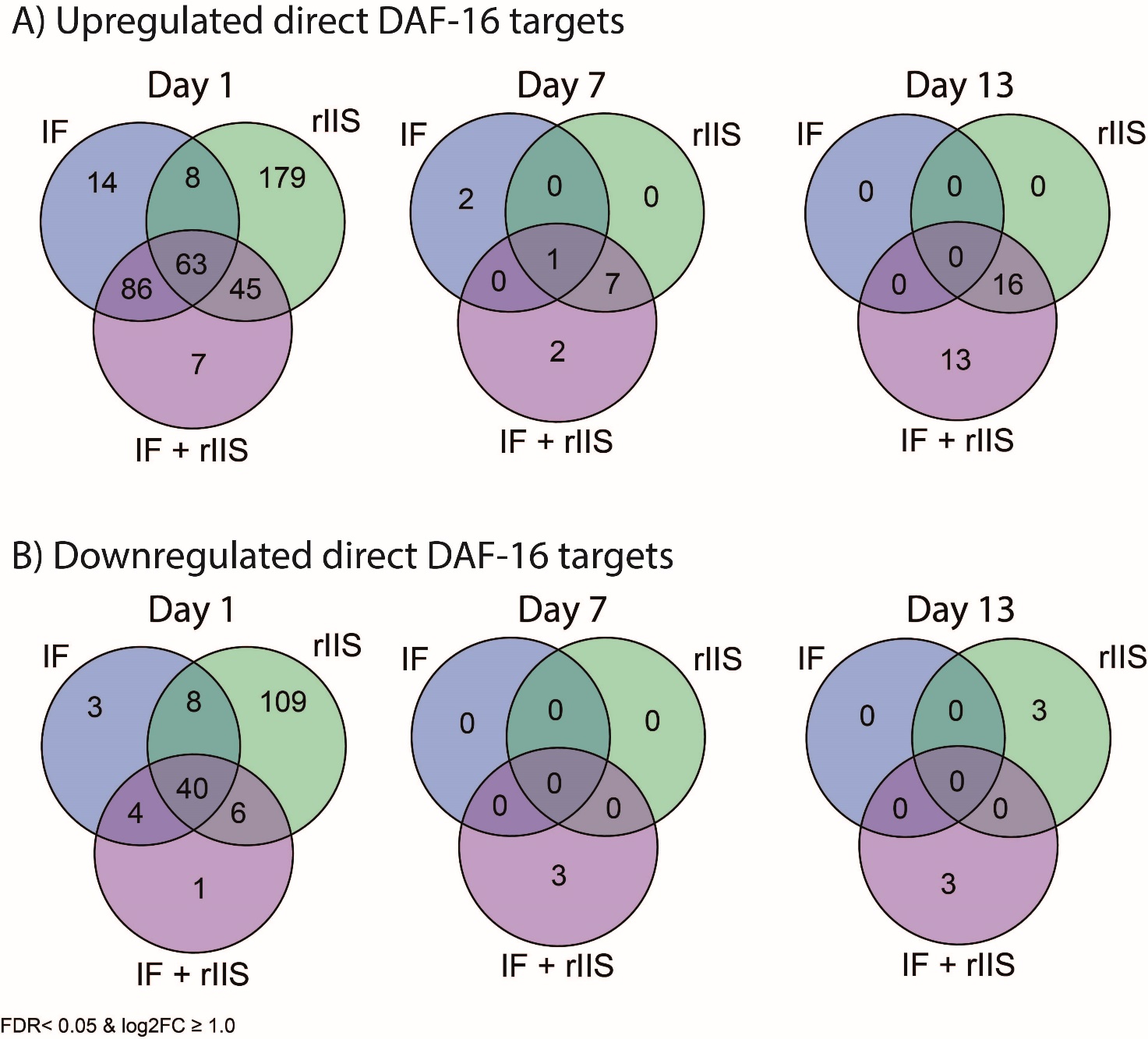


**Fig. S9.** Differentially regulated direct DAF-16 targets across IF, rIIS and the IF + rIIS treatments. Venn diagrams showing the number of direct DAF-16 targets that are upregulated (A) and downregulated (B) across IF, rIIS and combined treatments compared to control.


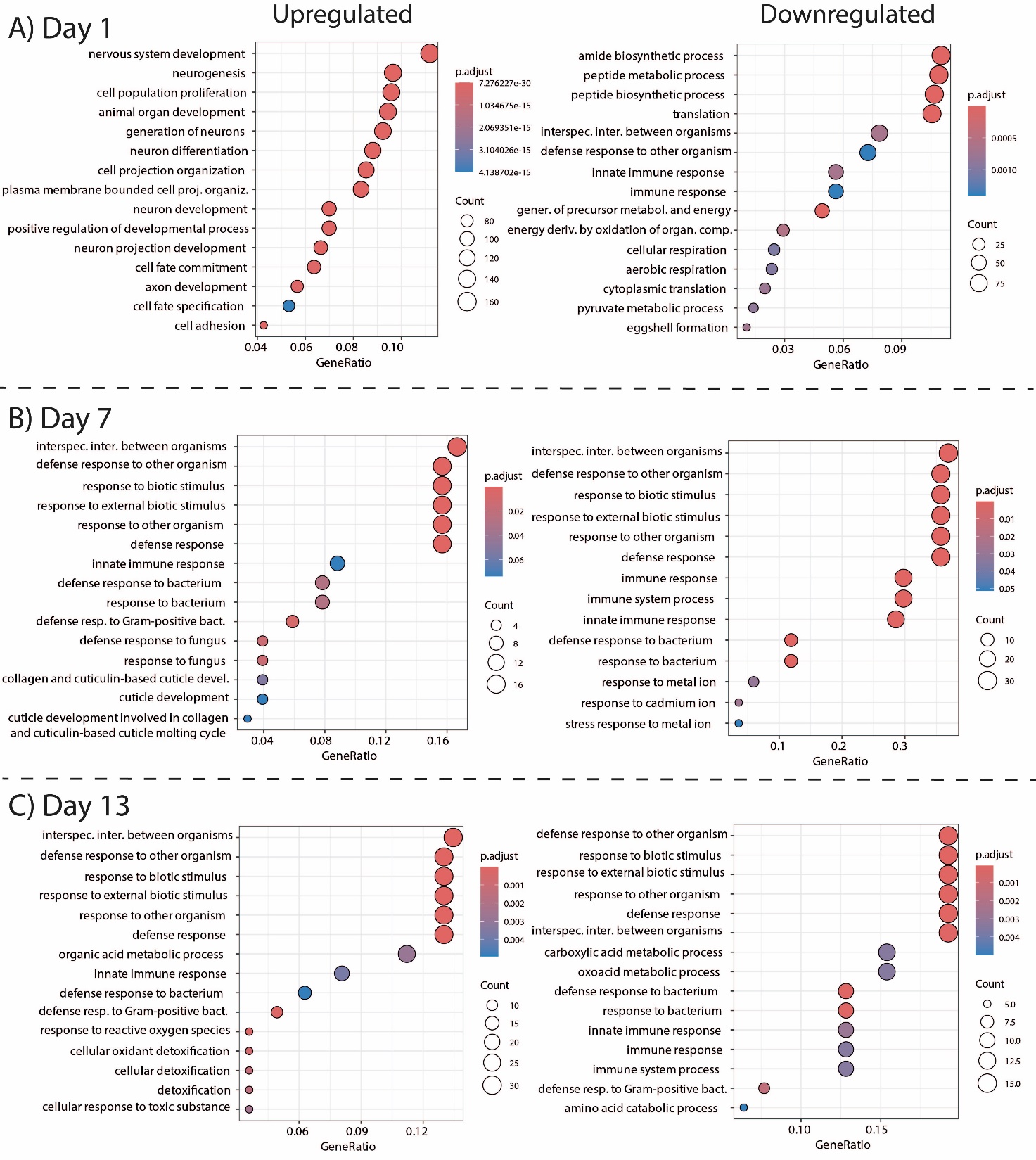


**Fig. S10.** Gene Ontology analysis of differentially expressed genes in the combined IF + rIIS treatment**.** GO term analyses showing the functions of genes up and down-regulated by the combined treatment compared to control across day 1 (A), day 7 (B) and day 13 (C).


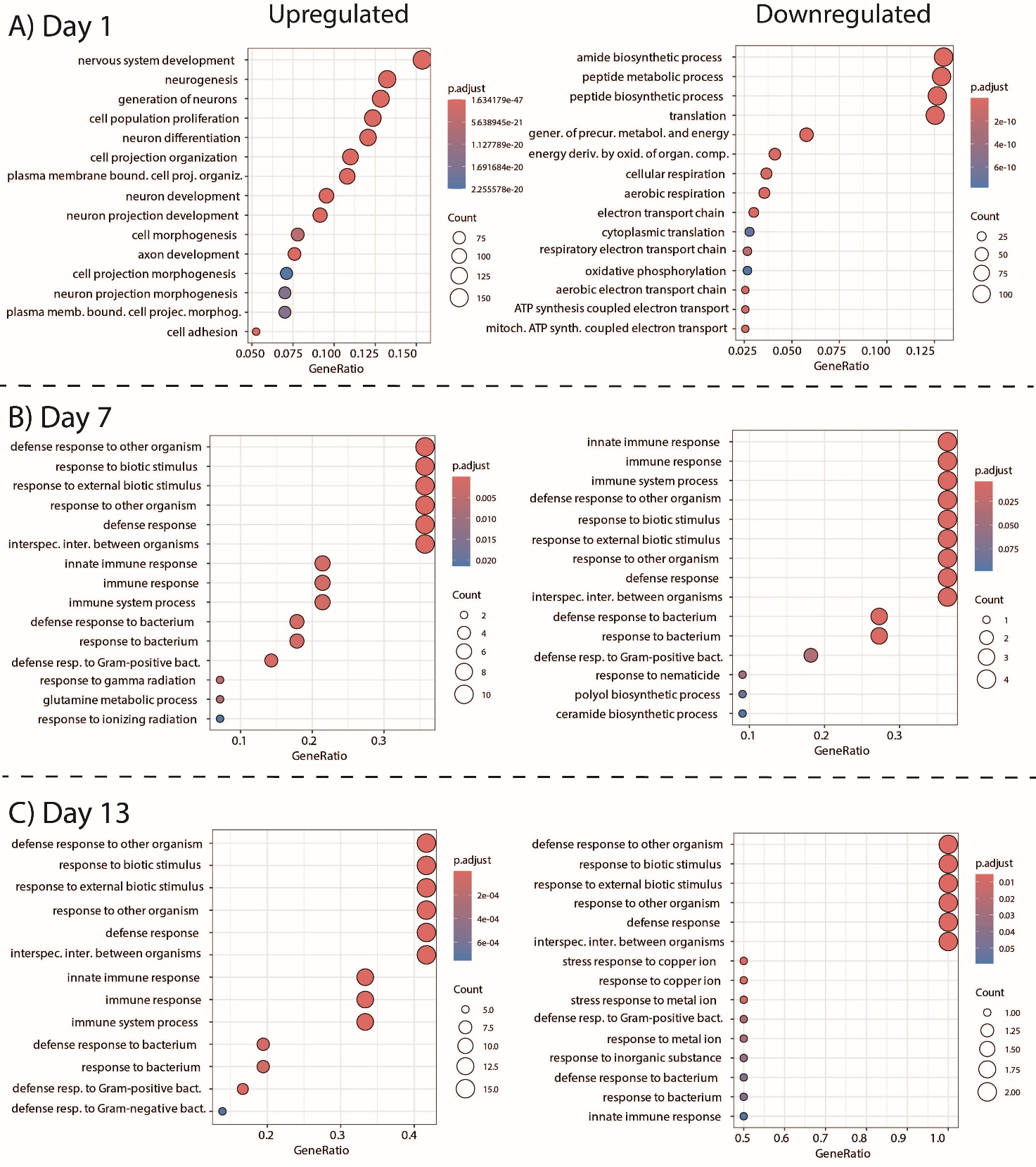


**Fig. S11.** Gene Ontology analysis of differentially expressed genes in the IF treatment. GO term analyses showing the functions of genes up and down-regulated by the IF treatment compared to control across day 1 (A), day 7 (B) and day 13 (C).


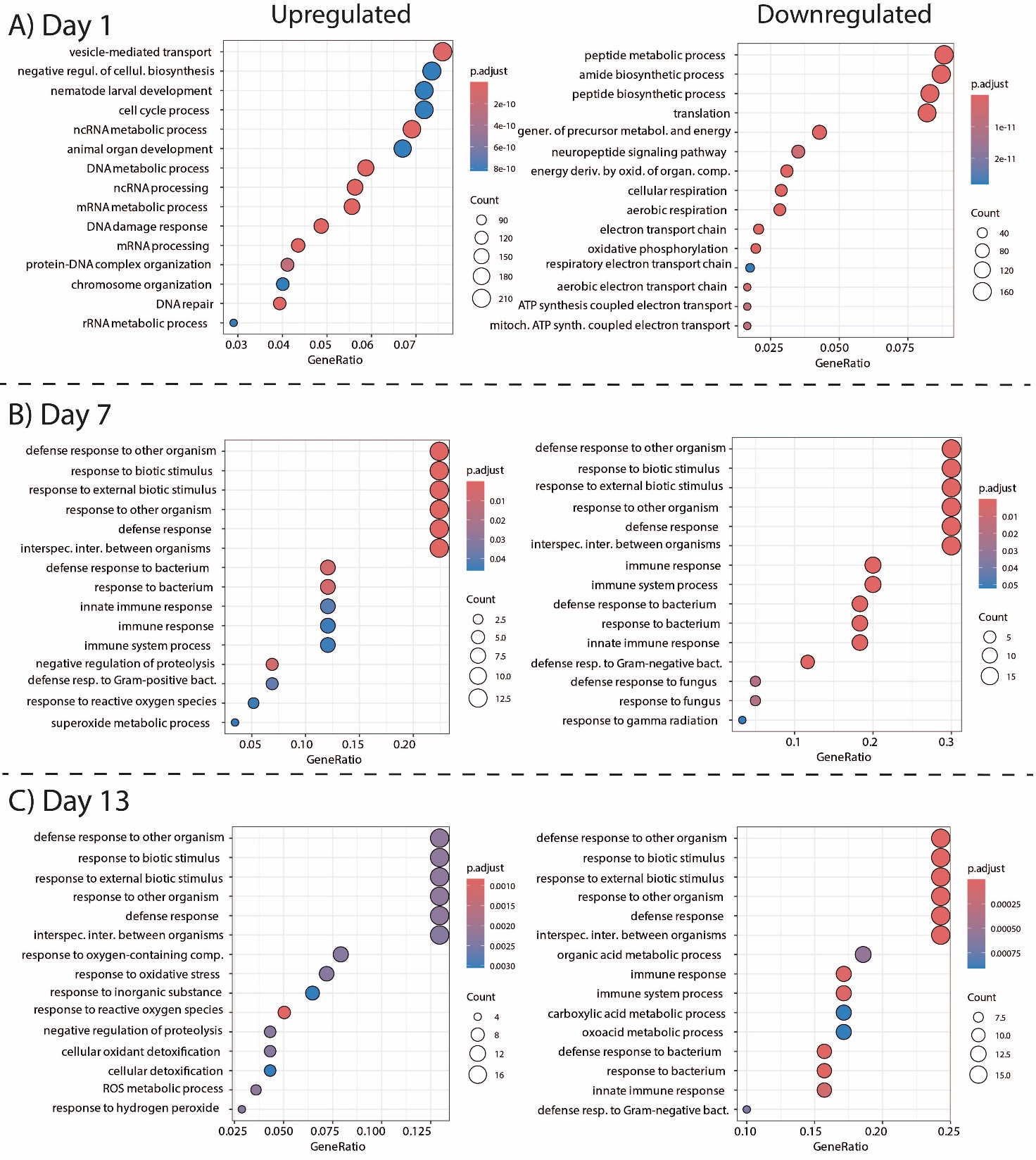


**Fig. S12.** Gene Ontology analysis of differentially expressed genes in the rIIS treatment. GO term analyses showing the functions of genes up and down-regulated by the rIIS treatment compared to control across day 1 (A), day 7 (B) and day 13 (C).


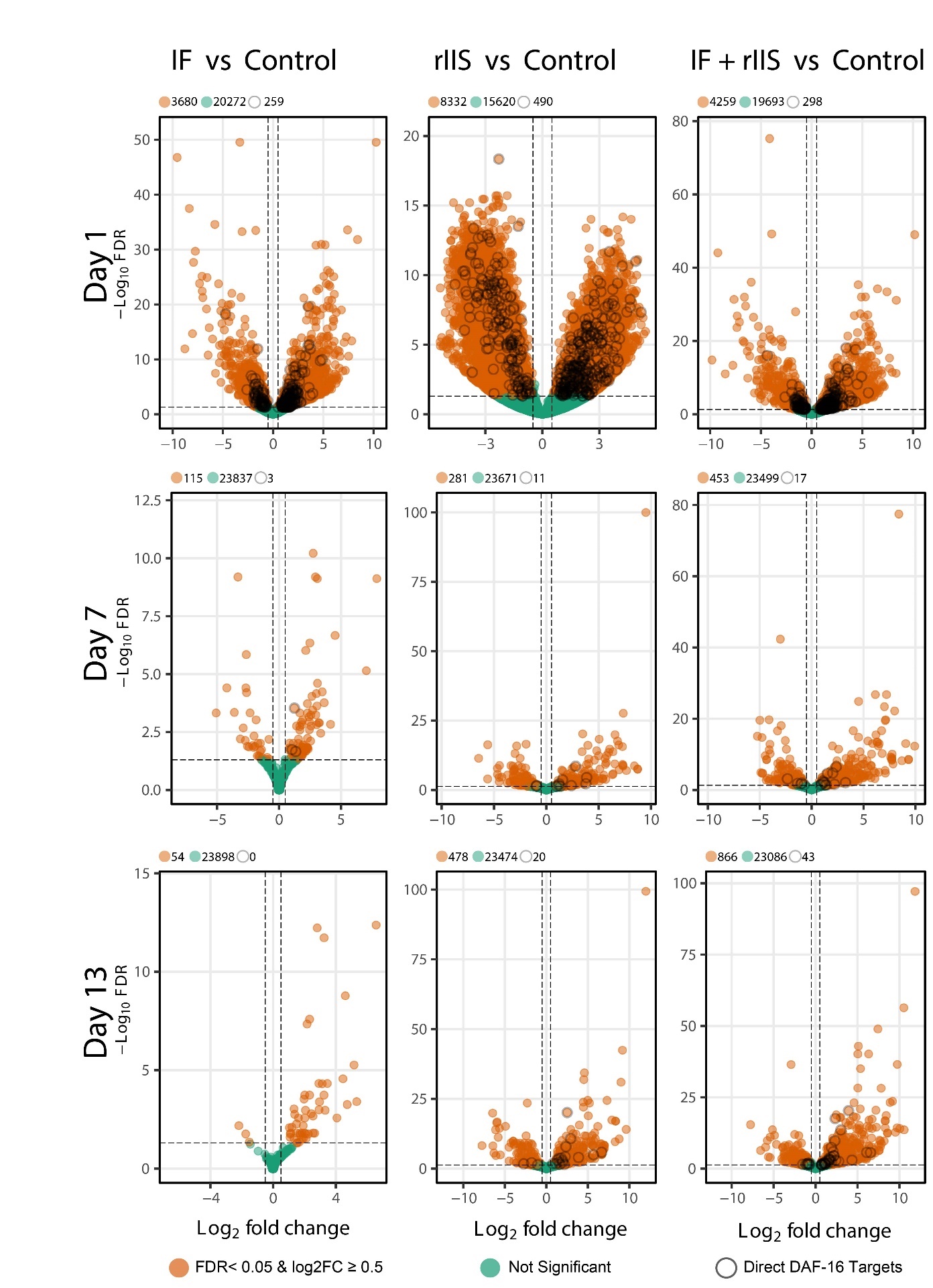


**Fig. S13.** Effect of IF, rIIS and IF + rIIS on age-specific gene expression (relaxed threshold: FDR< 0.05 & log2FC ≥ 0.5). Volcano plots comparing differentially expressed genes in IF, rIIS and IF + rIIS treatments compared to control on days 1, 7 and 13. DAF-16 direct targets were taken from a previous study that identified them by using chromatin profiling by DNA adenine methyltransferase identification, DamID.


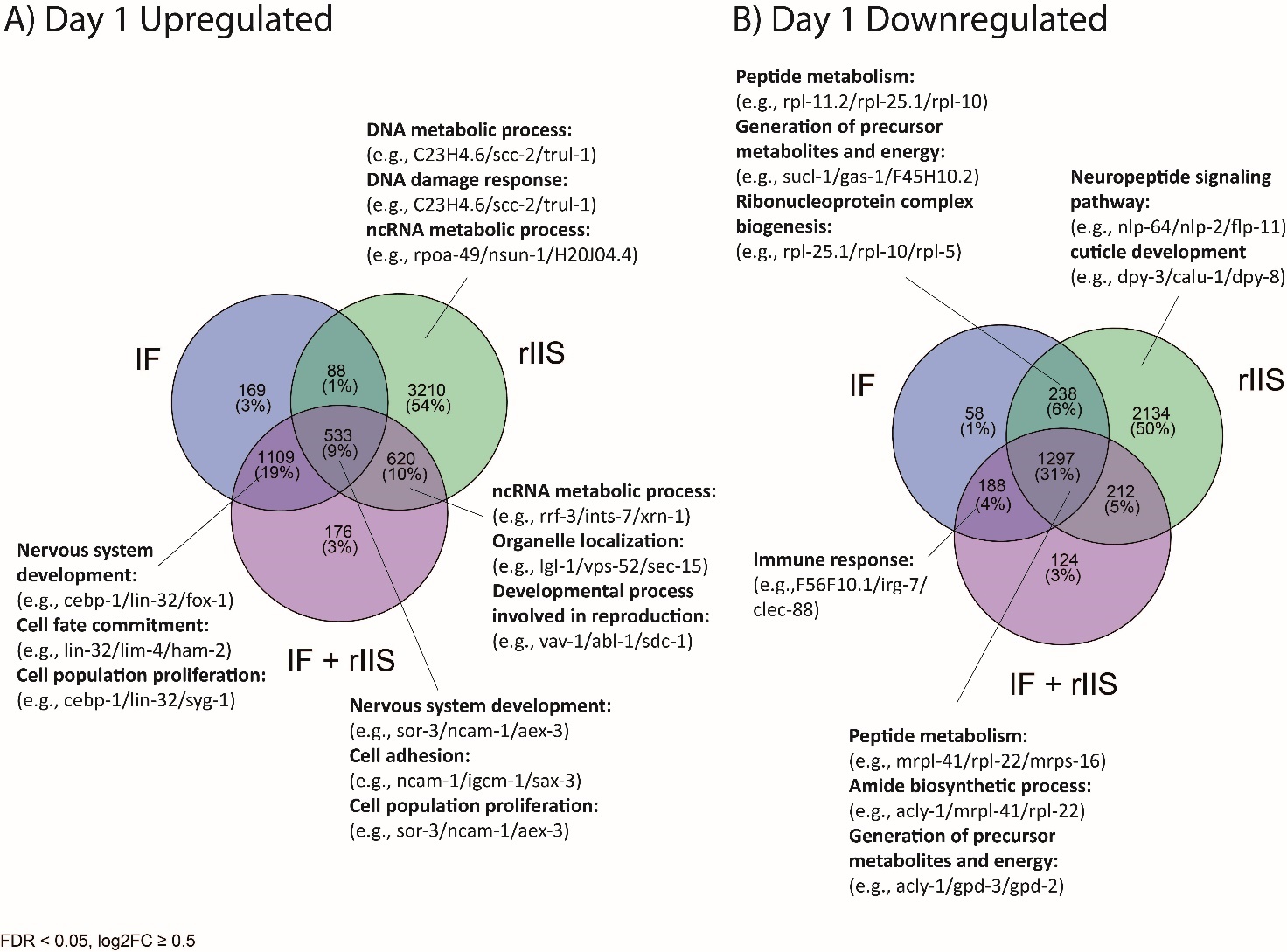


**Fig. S14.** Gene Ontology analysis of differentially expressed genes across IF, rIIS and combined treatments on day 1 (relaxed threshold: FDR< 0.05 & log2FC ≥ 0.5). GO term analyses showing the functions of genes up-regulated (A) and down-regulated (B) by IF, rIIS, and combined treatments compared to the control on day 1. The top three GO terms with the lowest adjusted p values are listed, each with up to three example gene names.


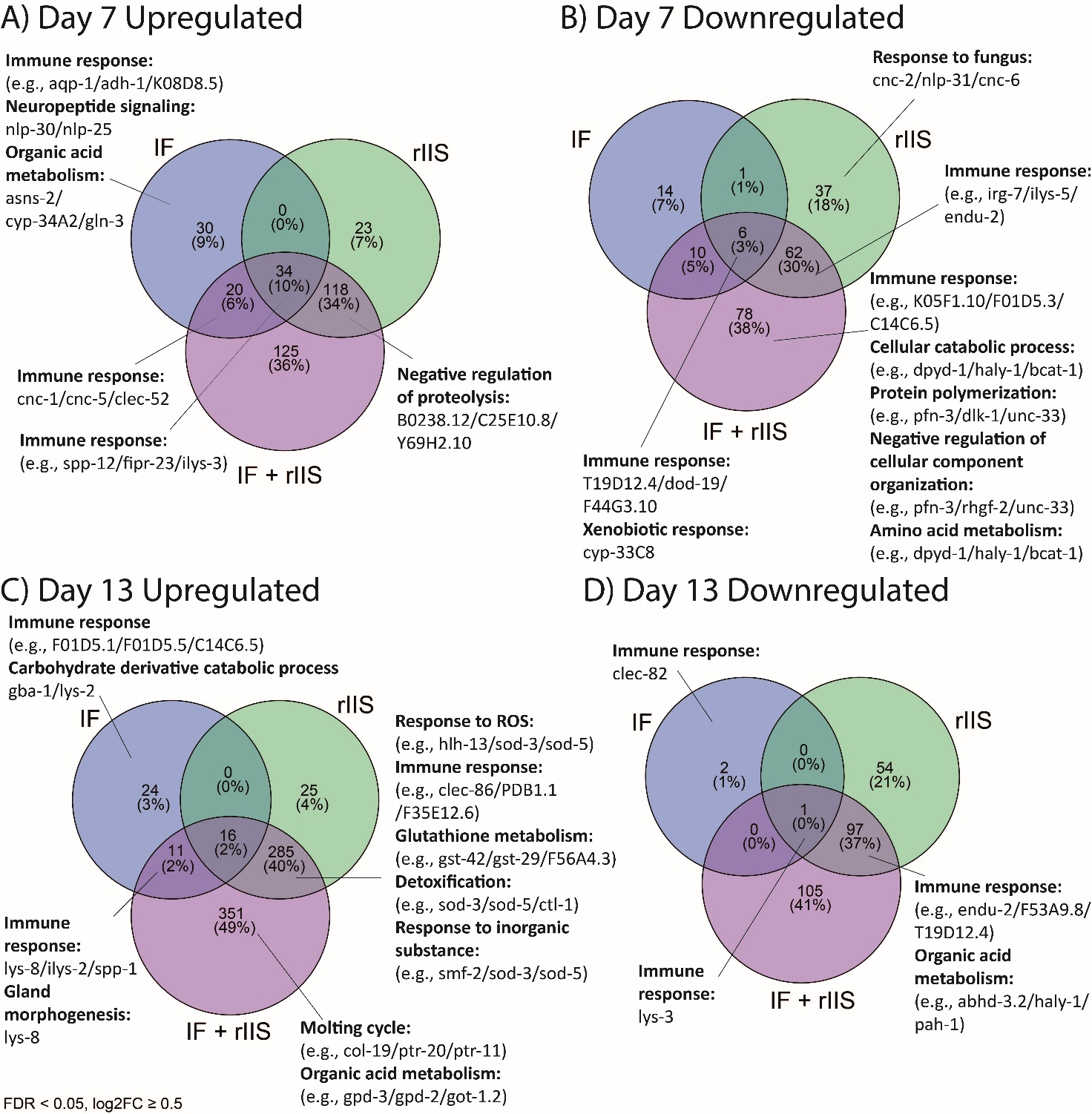


**Fig. S15.** Gene Ontology analysis of differentially expressed genes across IF, rIIS and combined treatments on day 7 and 13 (relaxed threshold: FDR< 0.05 & log2FC ≥ 0.5). GO term analyses showing the functions of genes up-regulated (A) and down-regulated (B) by different treatments on day 7; and up-regulated (C) and down-regulated (D) by different treatments on day 13. The top GO terms with the lowest adjusted p values are listed, each with up to three example gene names.

# Supplementary Methods

**Reproductive Assays**

Fore reproduction experiments, the hatched larvae were killed at 42°C for 3.5 hours and stored for counting later (Lind et al., 2019). Notably, the self-reproduction data for N2 population (but not USA and Portugal populations) presented in this study is also presented in our previous study (Duxbury et al., 2022), but in a different context. For mating experiments, males were produced by exposing around 50 L4 hermaphrodites to 30°C heat shock for 5 hours on NGM plates and then incubating them at 20°C (Aprison & Ruvinsky, 2015).

**Visualisation of DAF-16 nuclear localisation**

The *zIs356* strain was used for DAF-16 nuclear localisation assays (Wolff et al., 2006). Worms were identified as having nuclear GFP if at least one nucleus displayed DAF-16-GFP localisation; otherwise, they were considered to have cytoplasmic GFP, following the method described by Wolff et al. (2006). The nuclear localisation was observed specifically in the head region of the worms as previously done by Weinkove et al. (2006).

**Data processing**

Standard adapter sequences, common contaminants were removed from the paired-end reads using BBduk from the BBtools package version 37.62 (Bushnell, 2023). The quality of the reads was assessed using FastQC version v0.12.1 and MultiQC version 1.13 (Andrews, 2010; Ewels et al., 2016). The reads were aligned to the WBcel235 *Caernohabditis elegans* reference genome using STAR version 2.7.8a (Dobin et al., 2013). Gene-level read counts were quantified using feature Counts version v2.0.3 (Liao et al., 2014).

**Differential expression analysis**

Differential gene expression analysis was performed using DESeq2 version 1.32.0 (Love et al., 2014). For log fold change, shrinkage with the "ashr" shrinkage estimator was used (Stephens, 2017).

**Gene Ontology analysis**

Gene Ontology enrichment analysis was performed using clusterProfiler version 4.2.0 (Wu et al., 2021) together with org.Ce.eg.db version 3.17.0 (Carlson, 2019).

**Statistical analysis**

We used the 'glmmTMB' package to fit generalized linear mixed-effects models using the 'glmmTMB' function, 'car' package to perform ANOVA analysis, 'ggplot2' package for data visualization, and 'MuMIn' package for model selection and inference (Barton & Barton, 2015; Fox et al., 2012; Magnusson et al., 2019; Wickham, 2011). All statistical analysis was done in R v. 3.6.2 (R Core Team, 2019)

**References**

Andrews, S. (2010). *FastQC: A quality control tool for high throughput sequence data*. https://www.bioinformatics.babraham.ac.uk/projects/fastqc/

Aprison, E. Z., & Ruvinsky, I. (2015). Sex Pheromones of C. elegans Males Prime the Female Reproductive System and Ameliorate the Effects of Heat Stress. *PLOS Genetics*, *11*(12), e1005729. https://doi.org/10.1371/journal.pgen.1005729

Barton, K., & Barton, M. K. (2015). Package ‘mumin.’ *Version*, *1*(18), 439.

Bushnell, B. (2023). *BBMap*. https://sourceforge.net/projects/bbmap/

Carlson, M. (2019). *org. Mm. eg. db: Genome wide annotation for Worm.*

Dobin, A., Davis, C. A., Schlesinger, F., Drenkow, J., Zaleski, C., Jha, S., Batut, P., Chaisson, M., & Gingeras, T. R. (2013). STAR: Ultrafast universal RNA-seq aligner. *Bioinformatics*, *29*(1), 15–21.

Duxbury, E. M. L., Carlsson, H., Sales, K., Sultanova, Z., Immler, S., Chapman, T., & Maklakov, A. A. (2022). Multigenerational downregulation of insulin/IGF-1 signaling in adulthood improves lineage survival, reproduction, and fitness in Caenorhabditis elegans supporting the developmental theory of ageing. *Evolution*, *76*(12), 2829–2845. https://doi.org/10.1111/evo.14640

Ewels, P., Magnusson, M., Lundin, S., & Käller, M. (2016). MultiQC: Summarize analysis results for multiple tools and samples in a single report. *Bioinformatics*, *32*(19), 3047–3048. https://doi.org/10.1093/bioinformatics/btw354

Fox, J., Weisberg, S., Adler, D., Bates, D., Baud-Bovy, G., Ellison, S., Firth, D., Friendly, M., Gorjanc, G., & Graves, S. (2012). Package ‘car.’ *Vienna: R Foundation for Statistical Computing*.

Liao, Y., Smyth, G. K., & Shi, W. (2014). featureCounts: An efficient general purpose program for assigning sequence reads to genomic features. *Bioinformatics*, *30*(7), 923–930. https://doi.org/10.1093/bioinformatics/btt656

Lind, M. I., Ravindran, S., Sekajova, Z., Carlsson, H., Hinas, A., & Maklakov, A. A. (2019). Experimentally reduced insulin/IGF-1 signaling in adulthood extends lifespan of parents and improves Darwinian fitness of their offspring. *Evolution Letters*, *3*(2), 207–216. https://doi.org/10.1002/evl3.108

Love, M. I., Huber, W., & Anders, S. (2014). Moderated estimation of fold change and dispersion for RNA-seq data with DESeq2. *Genome Biology*, *15*, 550. https://doi.org/10.1186/s13059-014-0550-8

Magnusson, A., Skaug, H. J., Nielsen, A., Berg, C., Kristensen, K., Maechler, M., van Bentham, K., Bolker, B. M., & Brooks, M. E. (2019). Generalized Linear Mixed Models using Template Model Builder. Package ‘glmmTMB’. *R Package Version 0.2.3*.

R Core Team. (2019). *R version 3.6. 2: A language and environmental for statistical computing*. Vienna, Austria: R Foundation for Statistical Computing.

Stephens, M. (2017). False discovery rates: A new deal. *Biostatistics*, *18*(2), 275–294. https://doi.org/10.1093/biostatistics/kxw041

Weinkove, D., Halstead, J. R., Gems, D., & Divecha, N. (2006). Long-term starvation and ageing induce AGE-1/PI 3-kinase-dependent translocation of DAF-16/FOXO to the cytoplasm. *Bmc Biology*, 4, 1-13. https://doi.org/10.1186/1741-7007-4-1

Wickham, H. (2011). Ggplot2. *Wiley Interdisciplinary Reviews: Computational Statistics*, *3*(2), 180–185. https://doi.org/10.1002/wics.147

Wolff, S., Ma, H., Burch, D., Maciel, G. A., Hunter, T., & Dillin, A. (2006). SMK-1, an essential regulator of DAF-16-mediated longevity. *Cell*, 124(5), 1039-1053. https://doi.org/10.1016/j.cell.2005.12.042

Wu, T., Hu, E., Xu, S., Chen, M., Guo, P., Dai, Z., Feng, T., Zhou, L., Tang, W., & Zhan, L. I. (2021). clusterProfiler 4.0: A universal enrichment tool for interpreting omics data. *The Innovation*, *2*(3), 100141.
